# Neuronal differentiation of neuroblastoma cell lines for neurological disease modeling

**DOI:** 10.1016/j.isci.2026.116469

**Published:** 2026-06-18

**Authors:** Claudia Pommerenke, Vivien Hauer, Sonja Eberth, Lisa Werr, Hannah Kallnischkies, Ulfert Rand, Stefan Nagel, Christoph Bartenhagen, Wilhelm Gerhard Dirks, Matthias Fischer, Laura Steenpass, Haicui Wang

**Affiliations:** 1 Department of Bioinformatics, IT, and Databases, Leibniz Institute DSMZ - German Collection of Microorganisms and Cell Cultures GmbH, 38124 Braunschweig, Germany; 2 Department of Human and Animal Cell Lines, Leibniz Institute DSMZ - German Collection of Microorganisms and Cell Cultures GmbH, 38124 Braunschweig, Germany; 3 Department of Experimental Pediatric Oncology, University Children’s Hospital of Cologne, 50931 Cologne, Germany; 4 Department of Translational Genomics, Faculty of Medicine and University Hospital Cologne, University of Cologne, 50931 Cologne, Germany

**Keywords:** Neuroscience, Molecular neuroscience, Transcriptomics

## Abstract

Neurological disorders are often associated with neuronal dysfunction. Neuroblastoma (NB) cell line SH-SY5Y is widely used as *in vitro* model as it can differentiate into neuron-like cells. Many other NB cell lines can also respond to differentiation treatment but their potential in neuronal modeling remained unexplored. We evaluated 18 NB cell lines using RNA-seq and differentiation treatment with retinoic acid and brain-derived neurotrophic factor. Only few mutations in neurotransmitter pathway genes were detected. Transcription factor activities and differentially expressed genes classified NB cell lines into adrenergic (ADRN) and mesenchymal (MES) types. Each ADRN-type cell line exhibited a unique expression profile of neurotransmitter pathway genes. Differentiation treatment of ADRN-type cell lines CHP-134, LAN-5, and SIMA resulted in neuron-like cells expressing synaptic marker and several neurotransmitter pathway genes. Our results revealed ADRN-type cell lines can be selected carefully based on molecular background, differentiation potential and neuronal gene expression for neurological disease modeling.

## Highlights

• Transcription factor activity inference with RNA-seq classifies ADRN- and MES-type
• Synaptophysin serves as an ADRN-type marker and neuronal differentiation marker
• Selected ADRN-type lines can form neuron-like cells by differentiation treatment
• Differentiated neuron-like cells express several neurotransmitter pathway genes

## Graphical abstract

**Figure:**
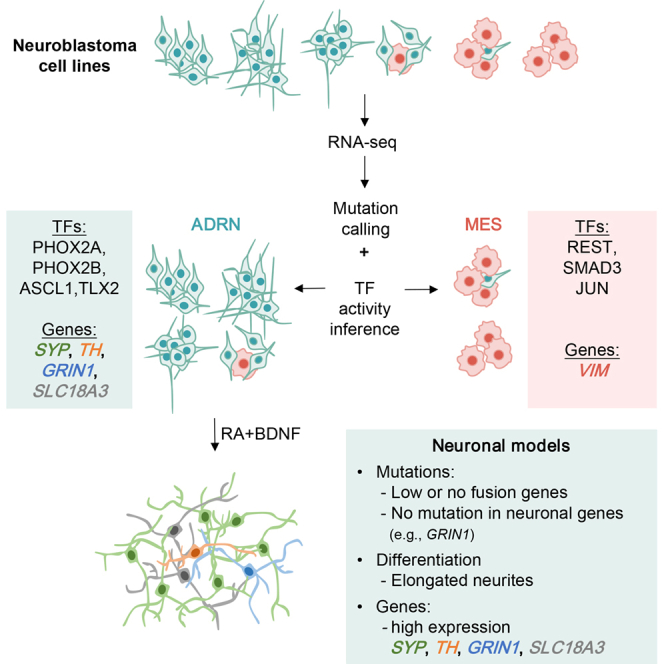

## Introduction

Neurons use neurotransmitters for synaptic communication. Neurons can be categorized into subtypes such as dopaminergic neuron, cholinergic neuron, glutamatergic neuron, GABAergic neuron, and serotonergic neuron by the neurotransmitters they produce and release. The disruption of particular subtype of neurons often leads to specific neurological disorders. It is known that the degeneration of dopaminergic neurons is a hallmark of Parkinson’s disease,^1^ and the degeneration and loss of cholinergic neurons is a major cause of Alzheimer disease.^2^ In addition, more recent next-generation sequencing (NGS) analyses identified a number of rare disorders caused by a single mutation in neurotransmitter pathway genes,^3^^,^^4^ raising the need of neuronal models expressing neurotransmitter pathway genes to explore the disease mechanism and treatment.

Neuronal models derived from immortalized cell lines offer a simple, defined option to investigate perturbed signaling pathways or impaired molecular function in relation to the pathophysiological mechanism,^5^^,^^6^^,^^7^ compared to the advanced animal models or induced pluripotent stem cell (iPSC)-derived neuronal models, which are often costly and technically demanding. Therefore, the human neuroblastoma (NB) cell line SH-SY5Y is widely used as a cost-effective, easily reproducible neuronal model,^5^^,^^6^^,^^7^ due to its capacity to form neuron-like cells upon treatment with retinoic acid (RA) and/or with additional compounds such as brain-derived neurotrophic factor (BDNF).^8^^,^^9^^,^^10^ The major and robust neuron subtype obtained from differentiation treatment by RA and BDNF in SH-SY5Y cells is cholinergic neuron.^9^^,^^10^ For other neuron subtypes, there were controversial reports for dopaminergic and glutamatergic neurons using RA or non-RA based differentiation protocols,^9^^,^^10^^,^^11^^,^^12^ consequently limiting the number of neurological disorders that can be modeled with the SH-SY5Y.

Similar to SH-SY5Y, many NB cell lines respond to RA treatment, making them potential candidates for neuronal models, though their response varies across different lines.^13^ This reflect the genetic diversity and cellular complexity of NB cell lines. The genetic diversity arises from the NB tumorigenesis through telomere maintenance mechanisms (TMMs)^14^ associated with activation of telomerase by *TERT* rearrangements and *MYCN* amplification, alternative lengthening of telomeres (ALTs), in some cases also with mutations in the *ALK* gene or in genes from the RAS/MAPK or MDM2/TP53 pathways.^15^^,^^16^ On the other hand, recent transcriptomic and epigenetic analyses (chromatin immunoprecipitation sequence [CHIP-seq]-based) defined two major cell types in NB cell lines, the neuronal adrenergic (ADRN) type associated with transcription factors (TFs) such as PHOX2A, PHOX2B, ASCL1, and neuronal markers such as tyrosine hydroxylase (TH) and dopamine-β-hydroxylase (DBH),^17^^,^^18^^,^^19^^,^^20^^,^^21^ and the non-neuronal mesenchymal (MES) type expressing markers such as intermediate filament vimentin (VIM), fibronectin (FN1), or collagen isoform COL1A1.^17^^,^^18^^,^^21^ The genetic diversity interconnected with cell types further influenced the differentiation outcome, which was the major challenge in developing neuronal models using NB cell lines as mainly the ADRN-type responded well to RA treatment.

In this study, we explored the potential of different NB cell lines as neuronal models. We made in-depth molecular characterization of 18 publicly available NB cell lines, by identifying mutations in NB genes such as *MYCN* and *ALK* by DNA or RNA sequencing (RNA-seq), and additional mutations in neurotransmitter pathway genes. To provide a feasible method without CHIP-seq to identify an ADRN-type cell line, we utilized the TF activity inference method with RNA-seq data and classified the cell lines into ADRN-type and MES-type. Importantly, we show how to generate neuron-like cells expressing synaptic marker and several neurotransmitter pathway genes from different ADRN-type cell lines with RA and BDNF treatment as a proof of concept. These results would enable disease modeling for different neurological disorders in the future from existing NB cell lines.

## Results

### Molecular background of NB cell lines

**Figure 1:**
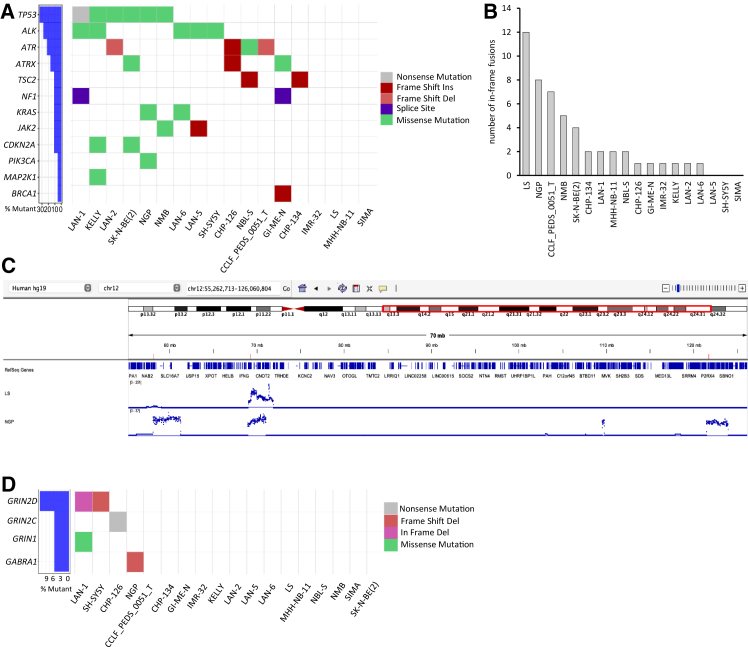
Mutations identified in neuroblastoma cell lines (A) Waterfall plot of recurrent non-synonymous mutations in NB cell lines called on RNA-seq data for NB disease-associated genes. Mutation recurrency was displayed as percentage (%) of mutated cell lines among 17 NB cell lines. (B) The number of in-frame gene fusions detected in RNA-seq data of NB cell lines predicted with at least one fusion calling algorithm and a minimal split read count of 2 (see also Table S3). (C) The cell lines LS and NGP showed amplifications in 12q. Screenshot from IGV browser of WGS data. (D) Waterfall plot of recurrent non-synonymous mutations in NB cell lines called on RNA-seq data for selected neurotransmitter pathway genes. Mutation recurrency was displayed as percentage (%) of mutated cell lines among 17 NB cell lines. See also Table 1 for summary information.

In order to gain insights into the molecular background of a panel of 18 NB cell lines (Table S1), their RNA was subjected to RNA-seq. Data were used for gene expression profiling and variant calling of key mutations in NB-related genes.^16^ Most mutations identified from gene transcripts were single-nucleotide polymorphisms (SNPs) as missense, nonsense, small indels, or splice site mutations, including frequent *TP53* mutations in 6 NB cell lines, *ALK* mutations in 5 NB cell lines, and mutations in other known NB genes (*ATR*, *ATRX*, *KRAS*) in more than two NB cell lines (Figure 1A; Table 1; Table S2). Genetic alterations including indels and translocations often lead to gene fusions, which can also be called from RNA-seq data.^22^ The recently reported *ALK::TERT* fusion gene was confirmed in the cell line KELLY^16^ (Table S3). LS carried the highest number of in-frame fusions (Figure 1B; Figure S1; Table S3) with most genes from chromosome 12, consistent with the observation of breakpoints and amplifications in chromosome 12, where cell line NGP also showed amplifications (Figure 1C).

**Table 1:** Key features of NB cell lines

cell line	*MYCN* copy number	*MYCN* expression	NB mutations	Genomic rearrangement	Fusion numbers (example)	Neuron gene mutations	TRAP
CCLF_PEDS_0051_T	2	0	*ATR* p.E797Rfs∗2	–	7	–	negative
CHP-126	533	++	*ATR* p.S1038Ffs∗11, *ATRX* p.R840Kfs∗9	–	1	*GRIN2C* p.Y551∗, *GRIN2C* p.V191L	positive
CHP-134	243	+++	*TSC2* p.V299Cfs∗39	–	2	–	positive
GI-ME-N	2	0	*NF1* p.×20_splice, *ATRX* p.V1181L	*NF1* and *TERT* rearrangement	1	–	positive
IMR-32	62	+++	–	–	1	–	positive
KELLY	210	++	*ALK* p.F1174L, *TP53* p.P177T	*TERT* rearrangement	1 (*ALK::TERT*)	–	positive
LAN-1	121	++	*ALK* p.F1174L, *TP53* p.C182∗, *NF1* p.×1526_splice	–	2	*GRIN1* p.L138M, *GRIN2D* p.Q1001_K1002del	positive
LAN-2	34	+	*ATR* p.K764E, *ATR* p.S749P, *ATR* p.F329Lfs∗14, *TP53* p.R337C	–	1	–	positive
LAN-5	223	+++	*ALK* p.R1275Q	–	0	–	positive
LAN-6	3	0	*ALK* p.D1091N, *KRAS* p.G12C	–	1	–	positive
LS	79	++	–	–	12 (*CNOT2::TSPAN8*)	–	positive
MHH-NB-11	87	++	–	–	2	–	positive
NBL-S	3	+	*ATR* p.Y2132D, *TSC2* p.V299Cfs∗39	–	2	–	positive
NMB	97	+++	*TP53* p.G245S	–	5	–	positive
NGP	138	+++	*TP53* p.C141W, *KRAS* p.M170V	–	8	*GABRA1* p.Y196Mfs∗13	positive
SH-SY5Y	2	0	*ALK* p.F1174L	–	0	*GRIN2D* p.H829Qfs∗2	positive
SIMA	54	+	–	–	0	–	positive
SK-N-BE(2)	314	+++	*TP53* p.C135F, *ATRX* p.R907Q, *ATRX* p.N755K	–	4	–	positive

Genetic alterations such as the amplification of the *MYCN* proto-oncogene is a hallmark of NB.^16^ Therefore, using droplet-based digital PCR (ddPCR) we verified *MYCN* amplification in 13 out of 18 NB cell lines on genomic DNA (Table 1; Figure S2). Other common NB-related genetic alterations such as *NF1* or *TERT* gene rearrangements were verified from whole genome sequencing (WGS) data, showing GI-ME-N with both *NF1* and *TERT* gene rearrangements (Table 1). In the KELLY cell line, rearrangement of the *TERT* locus was also confirmed experimentally by fluorescence *in situ* hybridization (FISH) analysis (Table 1). The *TERT* rearrangement, high *MYCN*, or *TERT* expression could activate telomerase activity in NB, thereby making the NB cell lines immortal.^14^ Using Fluorescent Telomeric Repeat Amplification Protocol (f-TRAP) assay, we showed 17 out of 18 NB cell lines positive for telomerase activity, leaving that one cell line, namely CCLF_PEDS_0051_T, might either be not immortal or not TMM positive (Table 1).

In addition to NB-associated mutations, it was essential to identify mutations from the neurotransmitter pathway genes in NB cell lines in order to use them as models for neurological disorders. A total of 17 neurotransmitter pathway genes associated with 5 major neurotransmitter-based neuron subtypes and also with known neurological disorders were selected for mutation calling (Table S4). Importantly, only four cell lines (CHP-126, LAN-1, NGP, and SH-SY5Y) including the commonly used SH-SY5Y carried mutations in the selected neurotransmitter pathway genes (Figure 1D; Table S5), making most NB cell lines mutation-free for these neurotransmitter pathway genes.

Taken together, our molecular characterization of the panel of NB cell lines provides an overview of the genetic background of each individual cell line. Although the NB cell lines gained immortality through diverse genetic mutations related to NB tumorigenesis, most of them are free of mutations in selected neurotransmitter pathway genes, meeting the basic requirement for modeling neurological disorders.

### TF activity inference to classify ADRN- and MES-type

**Figure 2:**
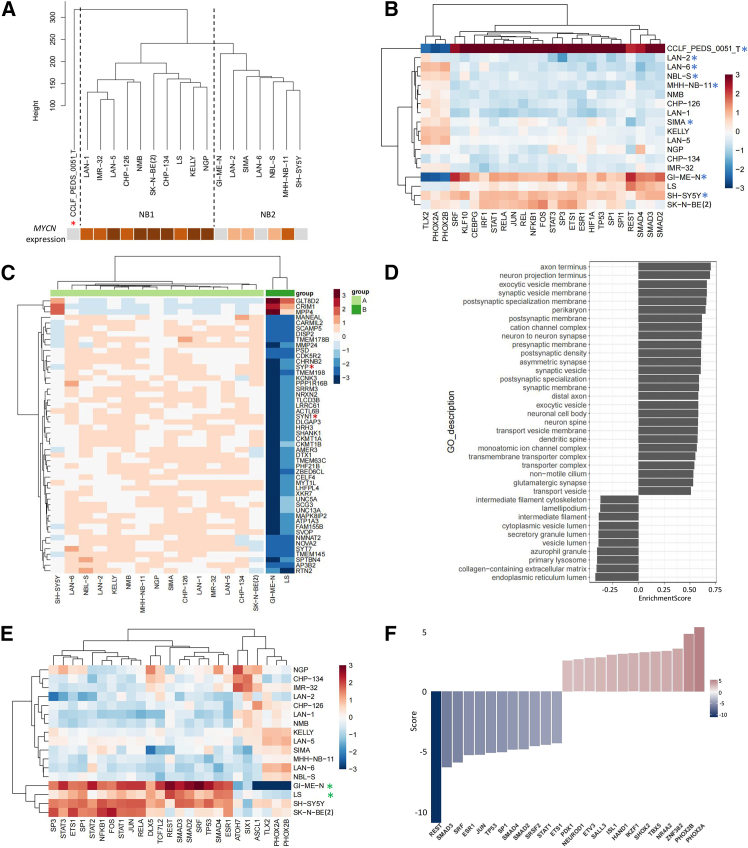
Differential gene expression and transcription factor activity of ADRN- and MES-type cell lines (A) Transcriptome-wide unsupervised clustering analysis separates NB cell lines into cluster NB1 and cluster NB2. NB1 correlated to *MYCN* high expression and NB2 correlated to *MYCN* low expression (see also Figure S3; Table 1 for *MYC* expression). ∗CCLF_PEDS_0051_T belongs to NB2 group with *MYCN* low expression. (B) TF activity heatmap depicting TF activity for regulating gene expression of NB1 and NB2 groups. The TF activity was inferred by decoupleR based on the collection of CollecTRI regulons with processed bulk RNA-seq data. Blue stars (∗) pointed to cell lines of group NB2. (C) Top 50 DEGs of assigned group A and group B according to activities of ADRN-type TF PHOX2A, PHOX2B, TLX2 and MES-type TFs such as SMAD3 from Figure 2B. Group A correlated to ADRN-type and group B correlated to MES-type. Red stars (∗) highlighted examples of two neuronal genes. (D) Gene set enrichment analysis (GSEA) for Gene Ontology (GO) of ADRN- and MES-type. (E) TF activity heatmap depicting TF activity for regulating ADRN- and MES-type cell lines. The TF activity was inferred by decoupleR based on the collection of CollecTRI regulons with processed bulk RNA-seq data. Green stars (∗) pointed to two possible MES-type cell lines (group B). (F) The top suppressed (blue) and activated (red) TFs linked to DEGs of ADRN- and MES-type. TF activities were inferred from the *t* values of the DEGs between ADRN- and MES-type (group A vs. group B) by decoupleR.

ADRN-type NB cells are considered better candidates for neuronal models compared to MES-type cells, as they typically exhibit a neuronal morphology and high sensitivity to treatment with RA.^23^ To distinguish the neuronal ADRN-type and non-neuronal MES-type, we performed comprehensive analysis of gene expression and TF activities using the generated RNA-seq data. Unsupervised clustering analysis revealed two major groups, NB1 and NB2 (Figure 2A). While NB1 correlated to *MYCN* high expression, NB2 was associated with low or absent *MYCN* expression including the CCLF_PEDS_0051_T, which was clustered separately from all other cell lines (Figure 2A; Figure S3). Analysis of differentially expressed genes (DEGs) between NB1 and NB2 cell lines (Figure S4A) and gene set enrichment analysis (GSEA) on Gene Ontology (GO) terms revealed upregulated genes involved in neuron signaling and neuron structural organizations, but downregulated genes in cytoskeleton, cellular matrix, and cell membranes (Figure S4B). Next, we explored the TF activity inference with overall gene expression changes between NB1 and NB2 cell lines, which revealed active TFs such as PHOX2A and PHOX2B and suppressed TFs including REST, SMAD2, SMAD3, and TP53 in most NB cell lines (Figure 2B). Therefore, the unsupervised clustering analysis revealed expression patterns of cell lines indicative for ADRN- or MES-type due to highly active ADRN TFs PHOX2A and PHOX2B^17^^,^^18^^,^^19^^,^^20^^,^^21^ in most cell lines, but active MES TFs such as SMAD3^17^^,^^18^^,^^21^ mainly in three cell lines (CCLF_PEDS_0051_T, LS, GI-ME-N) (Figure 2B).

To verify the ADRN and MES identity, we assigned the PHOX2A and PHOX2B active cell lines to group A and two cell lines GI-ME-N and LS to group B as both had inactive TFs PHOX2A and PHOX2B but active TF SMAD3. Based on this definition, CCLF_PEDS_0051_T belonged to group B as well, but it was excluded from further analyses due to its extremely high TF activities compared to all other NB cell lines (Figure 2B). The group A had significant enrichment of many neuron functional genes such as *SYP* and *SYN1* (Figure 2C; Table S6), which encode synaptophysin (SYP) and synapsin 1 (SYN1) protein respectively, two synaptic vesicle proteins essential for neurotransmission and neuronal development.^24^ GSEA confirmed upregulated genes involved in either neuron signaling or neuron structural organizations but downregulated genes involved in non-cell type-related general organelles or cellular cytoskeleton (Figure 2D; Table S7). Further TF activity inference revealed that one TF set consisting of PHOX2A, PHOX2B, TLX2, ASCL1 was exclusively active in group A, while the other TF set comprising ESR1, SMAD2, SMAD3, SMAD4, REST, JUN, TP53 was strongly active in group B (Figure 2E). However, we also observed the active status of both TF sets in two cell lines SH-SY5Y and SK-N-BE(2) from group A (Figure 2E), suggesting mixed features of these two cell lines. Among the top activated and deactivated TFs regulating DEGs between group A and B, PHOX2A and PHOX2B were the highly activated and REST was the top deactivated (Figure 2F).

In summary, the group A of NB cell lines was enriched for expression of neuronal genes in undifferentiated state, associated with a set of TFs including the two known ADRN TFs PHOX2A and PHOX2B, strongly suggesting this group of cell lines to be ADRN-type. The group B was associated with another set of TFs and non-neuronal genes for organelles and cytoskeleton, fitting to the definition of MES-type. In cell lines SH-SY5Y and SK-N-BE(2), both TF sets for ADRN- and MES-type were active, indicating the existence of cellular heterogeneity. Collectively, TF activity inference method with RNA-seq data was able to identify an ADRN-type cell line, revealing associated ADRN-type TFs without involving additional CHIP-seq, which provides a feasible classification method with available RNA-seq data.

### Differential expressed genes serve as ADRN- and MES-type markers for classification

**Figure 3:**
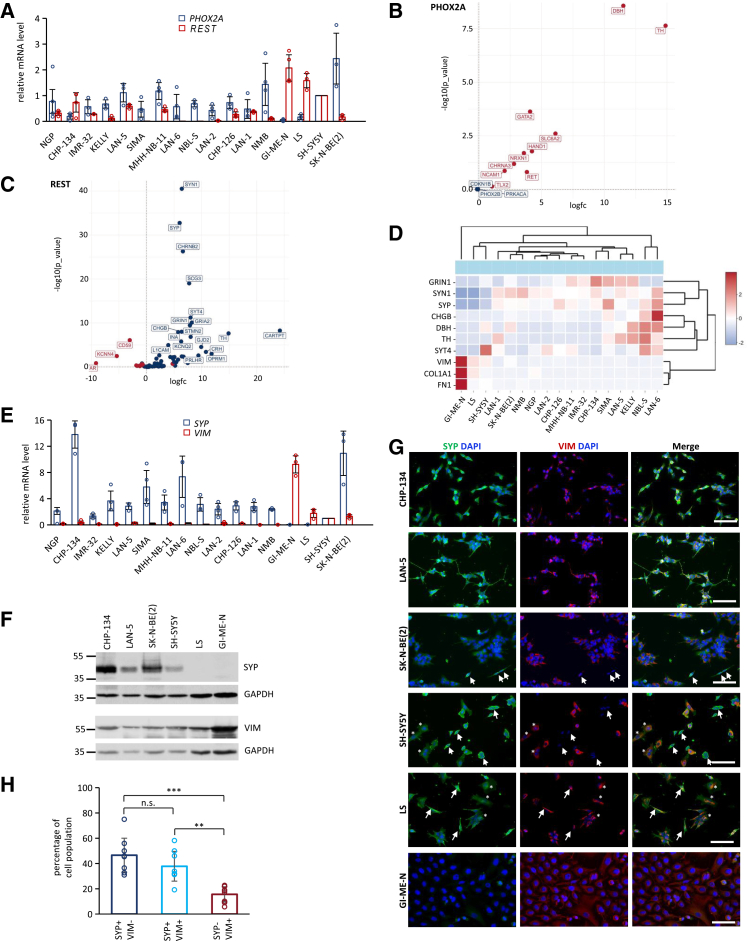
Differentially expressed genes as ADRN- and MES-type markers for classification (A) The gene expression level of *PHOX2A* (blue) and *REST* (red) as top ADRN- or MES-type TFs. The relative fold change detected by qPCR in different NB cell lines was reported as the level of mRNA relative to the SH-SY5Y cell line. Results are means ± SD (biological replicates, *n* ≥ 3). Each data point represents an independent biological replicate. (B) Predicted target genes being activated (red) and deactivated (blue) by the TF PHOX2A using decoupleR based on the collection of CollecTRI regulon. Most differential target genes in each TF along their *p* values were visualized in scatterplot. (C) Predicted target genes being activated (red) and deactivated (blue) by the TF REST using decoupleR based on the collection of CollecTRI regulon. Most differential target genes in each TF along their *p* values were visualized in scatterplot. (D) Expression heatmap of selected DEGs as ADRN- or MES-type markers. Figure was generated with DSMZCellDive using normalized expression from processed RNA-seq data. (E) The gene expression level of *SYP* (blue) and *VIM* (red) as ADRN- or MES-type markers. The relative fold change detected by qPCR in different NB cell lines was reported as the level of mRNA relative to the SH-SY5Y cell line. Results are means ± SD (biological replicates, *n* ≥ 3). Each data point represents an independent biological replicate. (F) Validation of selected ADRN-type marker SYP and MES-type marker VIM using western blot in selected NB cell lines. GAPDH served as loading control. (G) Cell morphology of ADRN- and MES-type cells. Cells were stained with ADRN-type marker SYP (green) and MES-type marker VIM (red). CHP-134 and LAN-5 represented ADRN-type and GI-ME-N represented MES-type cell lines. In SK-N-BE(2), SH-SY5Y and LS, arrows indicated high SYP signal and stars pointed to low SYP signal cells. Scale bars, 100 μm. (H) Quantification of different cell populations for cell line SH-SY5Y with 8 images captured from two independent experiments (4 images per experiment, with 30–60 cells per image). Each cell population (SYP+/VIM−, SYP+/VIM+, SYP−/VIM+) was presented as percentage of the total number of cells from each image and illustrated as one data point. Results are means ± SD (*n* = 8 images from two independent experiments). The comparison between multiple groups was performed using one-way ANOVA followed by Holm-Sidak’s pairwise multiple comparisons, ∗*p* < 0.05, ∗∗*p* < 0.01, ∗∗∗*p* < 0.001, n.s., no significant difference.

We next tested the possibility of using the regulatory TFs as markers to distinguish the ADRN- or MES-type NB cell lines. The high active TF PHOX2A from ADRN-type and REST from MES-type were selected. Quantification of gene expression using qPCR revealed relative high *REST* and low *PHOX2A* level in two MES-type cell lines, while *PHOX2A* level was higher in most ADRN-type than MES-type cell lines (Figure 3A). In principle, the mRNA expression of *PHOX2A* and *REST* could provide evidence for ADRN- or MES-type; however, it can only serve as an indicator as TF activity is not determined by expression level only.

We then explored to use DEGs as ADRN- and MES-type markers. In previous studies, *TH* and *DBH* were used as markers for the ADRN-type and *VIM*, *FN1*, *COL1A1* were used as markers for the MES-type,^21^ which were also among DEGs identified in our study (Table S6). In addition, by analysis of neuronal genes associated with ADRN TF PHOX2A and MES TF REST, we identified possible ADRN markers, which were neuronal genes *TH*, *DBH* likely positively regulated by PHOX2A (Figure 3B), or neuronal genes *SYN1*, *SYP*, *SYT4*, *GRIN1*, *CHGB*, *TH* likely negatively regulated by REST (Figure 3C). Therefore, we were able to demonstrate that 7 selected neuronal genes (*TH*, *DBH*, *SYN1*, *SYP*, *SYT4*, *GRIN1*, *CHGB*) were sufficient to identify the ADRN-type cell lines and 3 previously reported MES markers (*VIM*, *FN1*, *COL1A1*) were only positive in GI-ME-N and LS (Figure 3D), correlating to our described global expression profiles of ADRN- and MES-type (Figures 2C and 2D) and previously reported ADRN- and MES-type markers.^21^

To further reduce the number of markers for ADRN- or MES-type, we tested the possibility of using only one marker for each cell type to distinguish them. Indeed, ADRN-type marker *SYP* was exclusively detected in all ADRN-type cell lines and MES-type marker *VIM* was highly expressed in two MES-type cell lines (Figures 3D and 3E). Similarly, using western blot, we confirmed the protein expression of SYP in ADRN-type cell lines and its absence in the two MES-type cell lines (Figure 3F; Figure S5A). Despite that VIM is an abundant intermediate filament protein for both cell types, the MES-type cell line GI-ME-N showed the highest level of VIM expression (Figure 3F; Figure S5B).

Since the TF activity suggested a cellular heterogeneity in the cell lines SH-SY5Y and SK-N-BE(2) (Figures 2B and 2E), we performed immunostaining using SYP and VIM antibodies to identify ADRN- or MES-type cell populations within the individual cultures (Figure 3G). In ADRN-type cell lines CHP-134 and LAN-5, we observed VIM signal near the cell body but SYP appeared in the cell body and also the neurites (Figure 3G), indicating their ADRN identity. In contrast, in the MES-type cell line GI-ME-N only exclusive VIM signal was visible in the flat spread large cells (Figure 3G). However, LS culture contained a small fraction of high SYP expressing cells indicating the existence of ADRN-type cells in this assigned MES-type cell line (Figure 3G). Moreover, in SH-SY5Y cell culture, the major population represented small cells highly expressing SYP but lacking VIM, while a small fraction (10%–20%) of VIM positive cells with low SYP signal was observed (Figures 3G and 3H), revealing the transition of some ADRN-type cells to MES-type cells. Of note, the weak signal of SYP in SK-N-BE(2) cells might result from large cell clusters with few extended neurites, but single, non-clustered SK-N-BE(2) cells were also SYP positive (Figure 3G; Figures S5C–S5E). Together with *SYP* expression detected by qPCR and western blot (Figures 3E and 3F), our observation in cell morphology supported that SH-SY5Y and SK-N-BE(2) were more like an ADRN-type, but LS was a less prominent MES-type.

In summary, our analysis of target genes for single ADRN- or MES-type TF could also reveal the ADRN-type specific markers, which confirmed the established lineage markers from literature.^17^^,^^18^^,^^19^^,^^20^^,^^21^ Moreover, the highly relevant ADRN-type marker SYP and the MES-type marker VIM could identify the possible co-existence of both ADRN- and MES-type cell populations in one culture, further emphasizing the intracellular heterogeneity in some NB cell lines.

### ADRN cell lines prove potential to be differentiated into neuron-like cells

**Figure 4:**
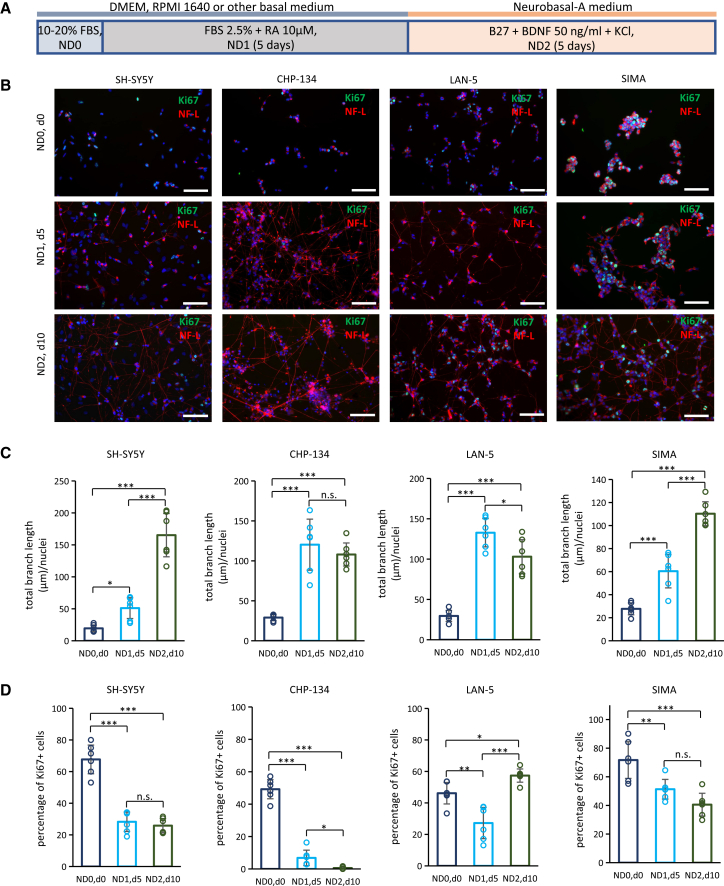
Neuronal differentiation of ADRN-type cell lines (A) Schematic representation of the three stages of neuronal differentiation (ND0, ND1, ND2) and applied media. (B) Immunostaining with proliferation marker Ki67 (green), and neuron-specific intermediate filament protein neurofilament light (NF-L) (red) for selected ADRN-type cell lines. Nuclei were stained with DAPI (blue). Scale bars, 100 μm. (C and D) Quantification of neurite length (C) or proliferation cell population (D) at different differentiation stages for 4 NB cell lines. For each cell line 6 images were captured from two independent experiments (3 images per experiment, each image contained more than 30 cells for undifferentiated stage, and more than 50 cells for differentiation stages). (C) Neurite length was presented as total branch length (μm)/nuclei and illustrated as one data point. Results are means ± SD (*n* = 6 images from two independent experiments). (D) Proliferation cell population Ki67+ was presented as percentage of the total number of cells from each image and illustrated as one data point. Results are means ± SD (*n* = 6 images from two independent experiments). The comparison between multiple groups was performed using one-way ANOVA followed by Holm-Sidak’s pairwise multiple comparisons, ∗*p* < 0.05, ∗∗*p* < 0.01, ∗∗∗*p* < 0.001, n.s., no significant difference.

To test the suitability of NB cell lines as models for neurological disorders, we tested their differentiation capacity into neuron-like cells. It is known from SH-SY5Y that ADRN-type cells can be induced to differentiate into neuron-like cells by treatment with RA (neuron differentiation stage 1, ND1), followed by BDNF treatment to promote neuronal maturation (neuron differentiation stage 2, ND2)^8^ (Figure 4A). Therefore, we applied this differentiation protocol using sequential treatment first with RA then BDNF to 15 ADRN classified cell lines from this study.

Under identical treatment conditions, the cell lines responded differently to RA and BDNF treatment, leading to a range of outcomes from high to low neuronal differentiation and survival (Figures S6A–S6D; Figure S7A). Some ADRN cell lines such as LAN-6, CHP-126, NBL-S, IMR-32 and MHH-NB-11 (Figure S6A) did not show strong differentiation after treatment, while another group including KELLY, LAN-1 and LAN-2 had a high differentiation grade but only few neuron-like cells survived (Figure S6B). Some ADRN cell lines (NMB, NGP, SK-N-BE(2)) largely maintained the clustering phenotype even after differentiation treatment, leading to heterogeneous neuronal phenotype (Figure S6C). Importantly, four ADRN cell lines (SH-SY5Y, CHP-134, LAN-5, SIMA) were converted to neuron-like cells with long neurites extending from the cell body (Figures 4B and 4C), enriched for neurofilament light chain (NF-L)—a common axonal marker. In contrast to differentiation, proliferation capacity decreased after RA treatment at ND1 and maintained at low proliferative status at ND2, whereas LAN-5 presented a significant recovery of cell growth at ND2 (Figures 4B and 4D).

**Figure 5:**
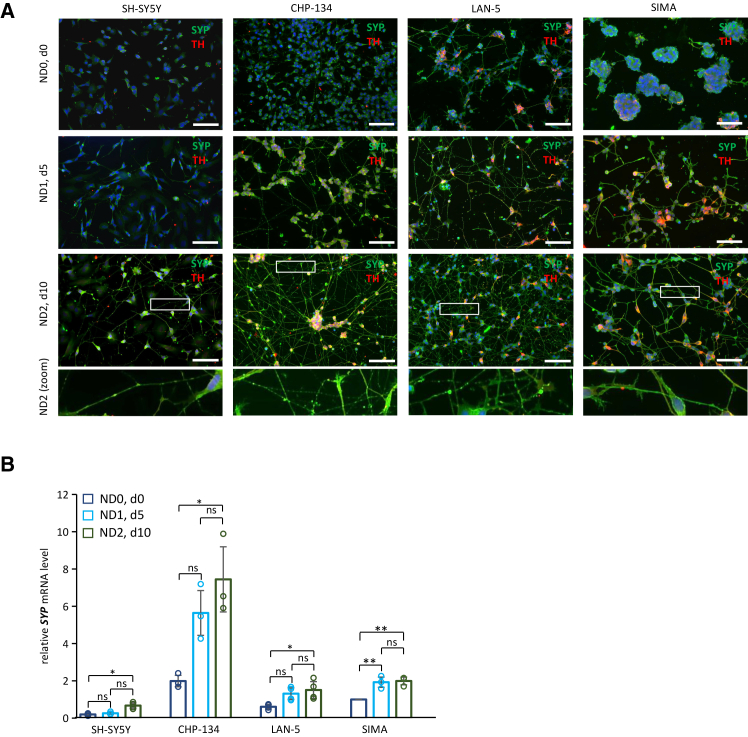
Synaptophysin serves as a neuronal differentiation marker (A) Immunostaining with synaptic vesicle marker SYP (green), and dopaminergic neuron marker TH (red) for selected ADRN-type cell lines. Nuclei were stained with DAPI (blue). Scale bars, 100 μm. The white frames at images of ND2, d10 were zoomed in to show the synaptic vesicles (bottom). (B) The *SYP* gene expression at endpoint of each neuron differentiation stage in selected NB cell lines. The relative fold change detected by qPCR in different NB cell lines was reported as the level of mRNA relative to the SIMA cell line of undifferentiated stage (ND0). Results are means ± SD (biological replicates, *n* ≥ 3). Each data point represents an independent biological replicate. The comparison between multiple groups was performed using one-way ANOVA followed by Holm-Sidak’s pairwise multiple comparisons, ∗*p* < 0.05, ∗∗*p* < 0.01, ∗∗∗*p* < 0.001, n.s., no significant difference.

The ADRN-type marker SYP was upregulated upon differentiation treatment (Figures 5A and 5B), which appeared as synaptic vesicle signals along neurite extensions during neuronal maturation (Figure 5A). In contrast, no neuron-like cells formed through differentiation induction and no SYP expression was detected in MES cell line GI-ME-N upon differentiation treatment (Figures S6D and S7D).

In summary, our data indicated that each cell line has different sensitivity toward the applied RA and BDNF differentiation protocol, leading to 3 ADRN-type cell lines (CHP-134, LAN-5, SIMA) capable of forming neuron-like cells in addition to SH-SY5Y. Moreover, SYP served as a suitable differentiation marker to track the neuronal differentiation.

### Neurotransmitter pathway genes expressed in ADRN-type cell derived neuron-like cells

**Figure 6:**
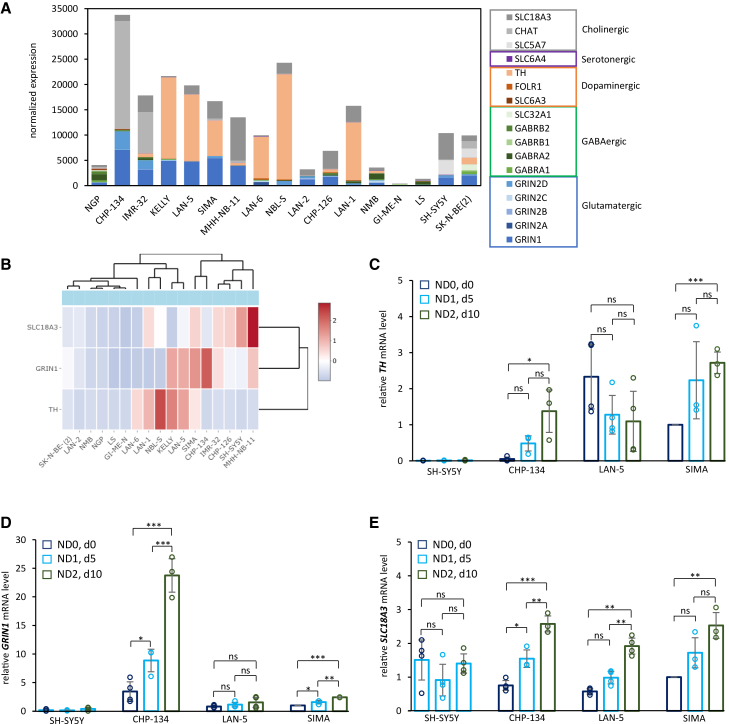
Neurotransmitter pathway genes expressed in ADRN-type cell derived neuron-like cells (A) Sum of gene expression of neurotransmitter pathway genes at undifferentiated stage (ND0) (color series—gray: cholinergic, purple: serotonergic, orange: dopaminergic, green: GABAergic, blue: glutamatergic). Normalized expression was obtained from processed RNA-seq data. (B) Expression heatmap of selected neuronal markers at undifferentiated stage (ND0). Figure was generated with DSMZCellDive using normalized expression from processed RNA-seq data. (C–E) Expression of selected markers (*TH*, *GRIN1*, and *SLC18A3*) at endpoint of each neuron differentiation stage in selected NB cell lines. The relative fold change detected by qPCR in different NB cell lines was reported as the level of mRNA relative to the SIMA cell line of undifferentiated stage (ND0). Results are means ± SD (biological replicates, *n* ≥ 3). Each data point represents an independent biological replicate. The comparison between multiple groups was performed using one-way ANOVA followed by Holm-Sidak’s pairwise multiple comparisons, ∗*p* < 0.05, ∗∗*p* < 0.01, ∗∗∗*p* < 0.001, n.s., no significant difference.

Next, we investigated the expression of neurotransmitter pathway genes in the ADRN-type cell derived neuron-like cells. Analysis of RNA-seq data from undifferentiated NB cells revealed that each ADRN-type cell line had preferential expression of neurotransmitter pathway genes already in the undifferentiated state, while MES-type cell lines (GI-ME-N and LS) lacked collective expression of these genes (Figure 6A). Only weak expression of GABAergic and serotonergic neuronal genes was detected in all cell lines. Among selected neurotransmitter pathway genes, *TH* and *GRIN1* were the main expressed dopaminergic and glutamatergic neuronal genes, respectively. Regarding the cholinergic neuronal genes, *SLC18A3* was widely expressed in ADRN-type cell lines but *CHAT* was highly enriched in CHP-134 and IMR32 and *SLC5A7* was mainly detected in SH-SY5Y and SK-N-BE(2). Overall, for 15 ADRN-type cell lines, dopaminergic neuronal gene expression was enriched in 6 ADRN-type cell lines, 3 of which (KELLY, LAN-5, SIMA) also depicted with high glutamatergic neuronal gene expression while another 3 cell lines (LAN-6, NBL-S, LAN-1) represented mainly dopaminergic neuronal gene expression. The other glutamatergic neuronal gene expressing cell lines (CHP-134, IMR-32, MHH-NB-11) had also high cholinergic neuronal gene expression. These data further reflected the cellular complexity of ADRN-type NB cell lines.

Therefore, we selected 4 representative ADRN-type cell lines (SH-SY5Y, CHP-134, LAN-5, SIMA) to investigate the neurotransmitter pathway gene expression after differentiation with RA and BDNF treatment. Additionally, three genes *TH*, *GRIN1*, *SLC18A3* were selected due to their broad coverage of most ADRN-type cell lines in the undifferentiated state (Figure 6B). Upon differentiation treatment, both CHP-134 and SIMA showed upregulation of *GRIN1*, *TH*, and *SLC18A3* (Figures 6C–6E), along with the SYP upregulation (Figure 5B), indicating the sequential maturation of neuron-like cells. Similar results were obtained from LAN-5 for *SYP* and *SLC18A3* but not for *GRIN1*, and *TH* expression which was high at undifferentiated stage dropped to a level similar to other cell lines after differentiation treatment (Figures 6C–6E). For SH-SY5Y, no *TH* expression and only slightly higher *GRIN1* expression were detected (Figures 6C and 6D).

In summary, we demonstrated the differentiation treatment of ADRN-type cell lines with RA and BDNF increased the expression of several neurotransmitter pathway genes, but to different extent for different ADRN-type cell lines. This may allow for modeling disease with specific affected genes such as *GRIN1* in epilepsy^4^ using cell line such as CHP-134 with relatively high *GRIN1* expression.

## Discussion

Neuronal models for neurological disorders must contain specific neuronal markers because most diseases often arise from the dysfunction or death of only one or a few specific neuron populations, like dopaminergic neurons in Parkinson’s, cholinergic neurons in Alzheimer disease^1^^,^^2^ or glutamatergic or GABAergic neurons in epilepsy^3^^,^^4^ (Table S4). Therefore, selecting adequate model systems with relevant neuronal gene expression is a crucial prerequisite for understanding of pathology and developing targeted therapies. In this study, we investigate the potential of 18 NB cell lines, including the commonly used SH-SY5Y, as neuronal models based on comprehensive molecular characterization and their neuronal differentiation potential, to assess their suitability as neuronal models.

We demonstrated that the majority of cell lines from our NB panel are ADRN-type, displaying neuronal-like phenotype and enriched for neuronal gene expression possibly controlled by active ADRN-type TFs PHOX2A, PHOX2B, ASCL1, HAND1, ISL1. The ADRN- or MES-type TFs that we identified using decoupleR, a Bioconductor package for TF activity inference from prior knowledge with bulk RNA-seq data, highly correlated to previous findings using ChIP-seq,^17^^,^^18^^,^^19^^,^^20^^,^^21^ suggesting a feasible and alternative method to identify ADRN-type TFs directly with RNA-seq data. REST was the predicted high active TF for MES-type cell lines and highly suppressed TF in ADRN-type cell lines from our analysis. Although REST was not identified as MES-type TF in previous reports,^17^^,^^18^^,^^21^ its enhanced activity was observed in more progressed clinical stages of NBs,^25^ which are often associated with the MES-type cells.^26^ It is not surprising that the assigned ADRN-type cell lines like SH-SY5Y and SK-N-BE(2) were associated with transcriptional activity related to both ADRN- and MES-type TFs, because interconversion can occur between ADRN- and MES-type cells during *in vitro* culture.^21^ Therefore, the analysis of global transcriptional activity combined with DEGs separates the MES-type from ADRN-type cell lines and also tracks any cellular heterogeneity within a single cell line.

We further extracted SYP as single marker for ADRN-type and VIM for MES-type to perform feasible identification of ADRN-type cell lines. The genes *TH* and *VIM* were frequently nominated as one of the major ADRN- or MES-type markers.^17^^,^^18^^,^^21^ However, *TH* gene expression was only detected in some ADRN-type cell lines of our NB panel. Alternatively, universal synaptic vesicle marker SYP or SYN^24^ was exclusively detected in all ADRN-type but not in MES-type cell lines of our NB panel. On the other hand, VIM was expressed in both ADRN- and MES-type cell lines, although it had relative higher expression level in MES-type than ADRN-type cells. With SYP and VIM as markers, we were able to classify the ADRN-type and MES-type cell lines. Moreover, SYP and VIM as markers were capable of detecting the cellular heterogeneity within one cell line in culture, allowing selection of an ADRN-type cell line with homogenous cells that can mature into uniform neuron-like cells.

While NB cell line SH-SY5Y is widely used for neuronal disease modeling,^5^^,^^6^^,^^7^ our study provides evidence for suitable alternative models such as CHP-134, SIMA and LAN-5 for neuronal modeling. The induced differentiation of ADRN-type cells by RA and BDNF was associated with increased SYP signal in clear synaptic vesicles, elongated neurites forming neuronal network, increased expression of neurotransmitter pathway genes, reassembling normal development from neural crest precursors to mature neurons,^27^ although the mature neuronal features such as electrophysiological properties and neurotransmitter release require further functional validation. At undifferentiated and differentiated status both CHP-134 and SIMA showed high glutamatergic and cholinergic neuronal gene expression. SIMA even showed additional high dopaminergic neuronal gene expression despite RA and BDNF do not favor the dopaminergic neuron formation.^10^ Therefore, the transcriptional profile of neurotransmitter pathway genes from the ADRN-type cell lines may provide a useful guide to select suitable NB cell lines for generating desired neuron subtypes. However, despite the neuron-like cells expressing some neurotransmitter pathway genes such as *TH*, *GRIN1*, and *SLC18A3*, transcriptomic profiling of differentiated samples to demonstrate coordinated pathway activation (in reference with our RNA-seq data from undifferentiated samples) is necessary to identify the neuron subtypes for precise disease modeling.

Previously published data showed that ADRN-type cell lines respond differently to RA and BDNF treatment, consistent with pre-clinical evaluation result of RA as a differentiation reagent for treatment, which revealed LAN-5 as most responsive and IMR-32 as hardly responsive cell lines.^13^ In this context, it would be important to transfer the testing results of other retinoids^13^ or non-retinoid chemicals such as Palbociclib^28^ as differentiation reagents from patient treatment to neuronal model generation. Additional consideration to improve differentiation output would be other compounds than RA and BDNF, such as staurosporine, a broad-spectrum protein kinase inhibitor, which significantly increased the formation of dopaminergic neurons of SH-SY5Y cells.^10^ Our NB panel consists of a number of high TH expressing ADRN-type cell lines, but their capacity to generate dopaminergic neurons requires future optimization with alternative compounds or adjusted differentiation medium.

We could not link the differentiation potential directly to key genomic features such as *MYCN* amplification status, *ALK* alterations. However, we did observe that overall mutation burden such as more fusion genes (in cell lines LS, NGP, NMB), or more genomic rearrangements (in GI-ME-N) associated with resistance to differentiation treatment. Despite the NB genetic background, only few cell lines from our NB panel carry one or two mutations in neurotransmitter pathway genes, allowing ADRN-type cell lines for modeling a large number of neurological disorders. However, a more in-depth analysis using multi-omics data are required to assure major disease pathways and modules are intact in the selected NB cell line in order to model individual neurological disorder.^29^ Besides, although here the applied fast differentiation protocol would allow high throughput screening using NB-derived neuronal models, the screening results would require further validation in neuronal models from high physiological relevant background such as iPSC-derived neurons to carefully avoid any potential influence from the tumor background of the NB cell lines.

Overall, this work provides comprehensive information on NB cell lines especially the genetic background of mutation status in neurotransmitter pathway genes, classification of ADRN- or MES-type using TF activity inference and DEG markers, expression profile of neurotransmitter pathway genes, and their differentiation potential. Furthermore, the proof of concept work using three alternative ADRN-type cell lines than SH-SY5Y to generate neuron-like cell models, may provide insights for disease modeling of neurological disorders using a broader portfolio of NB cell lines in the future.

### Limitations of this study

One major limitation is that we used a differentiation protocol optimized for SH-SY5Y cell line^8^ for all ADRN-type cell lines. Although we were able to show several well responsive cell lines for neuronal models, other cell lines might still hold the potential to differentiate well into neuron-like cells if the protocol can be adjusted individually, by optimizing seeding density, compound concentrations, and even the cell growth surface material and coating. So, no generalized conclusion was drawn for the differentiation potential. Therefore, although this study included 11 cell lines from male patients and 7 cell lines from female patients, sex-based effects were also not possible to be assessed as only 4 well responsive cell lines with this defined differentiation protocol were fully characterized.

Additionally, we had a limited number of MES-type cell lines in our NB panel compared to the large number of ADRN-type cell lines. The understanding of ADRN- and MES-type expression profiling could be improved by integrating more MES-type cell lines for analysis.

Technically, the evaluation of untreated and differentiation treated cells mainly relied on immunostaining-based quantification. The immunostaining-based intensity measurements in this study were performed with images captured from defined regions due to the uneven distribution of cells on cell culture surface (Figure S7C) and processed with software, which provided more qualitative (negative/positive as in Figures 3H and 4) than overall quantitative information. It will be important to further establish high throughput automated imaging and analysis with machine learning to obtain more quantitative and consistent results, and use other quantitative methods such as flow cytometry, western blot for further validation.

## Resource availability

### Lead contact

Further information and requests for resources and reagents should be directed to and will be fulfilled by the lead contact, Dr. Haicui Wang (haicui.wang@dsmz.de).

### Materials availability

This study did not generate new unique reagent.

### Data and code availability

• All data supporting the findings of this study are available within the article and its supplementary files. Additional raw data are available from the lead contact upon request.
• Raw and processed RNA-seq data have been deposited at ArrayExpress: E-MTAB-14737. Additionally, RNA-seq data are accessible at DSMZCellDive (https://celldive.dsmz.de/) for interactive visualization.
• WGS data for cell lines have been deposited at European Nucleotide Archive: PRJEB45367.^30^
• This study did not generate or use any custom code.

## Acknowledgments

We are grateful to Ida Steinkirchinger, Barbara Beuerle, Silke Fähnrich, Corinna Meyer, Maren Kaufmann, Anne Leena Koelz, Yvonne Merkhoffer, and Louis Graeber for technical support. This work was supported by the 10.13039/501100001659German Research Foundation (10.13039/501100001659DFG, 10.13039/501100001659Deutsche Forschungsgemeinschaft) as part of SFB1399 (grant ID 413326622 to M.F. and L.W.), SFB1588 (grant ID 493872418 to M.F.), and FI 1926/2-1 (to M.F.). The study was also supported by the Förderverein für krebskranke Kinder e.V. Köln (endowed chair to M.F.), Leverkusen hilft krebskranken Kindern e.V. (to M.F.) and the Bruno und Helene Jöster Stiftung (to M.F.).

## Author contributions

C.P., RNA-seq analysis; V.H. and H.W., cell culture, neuron differentiation, sample collections, western blot, and immunofluorescence; S.E., project conception, fusion gene analysis and validation; L.W., C.B., and M.F., WGS analysis; H.K., H.W., and V.H., qPCR; U.R. and S.N., *MYCN* gene copies and expression; W.G.D., TRAP assay; H.W., project conception, experimental design and project supervision; H.W., C.P., S.E., and L.S., manuscript writing.

## Declaration of interests

Authors except L.W., C.B., and M.F. are employed at the Leibniz-Institute DSMZ, a non-profit institute of the Leibniz Association, which distributes the cell lines used in this study for non-commercial use.

## STAR★Methods

### Key resources table

**Table:** 

REAGENT or RESOURCE	SOURCE	IDENTIFIER
**Antibodies**		

Anti-N-MYC, rabbit polyclonal	Cell Signaling	Cat# 9405; RRID: AB_10692664
Anti-GAPDH, mouse monoclonal	Abcam	Cat# ab8245; RRID: AB_2107448
Anti-Ki67, mouse monoclonal	BD	Cat#550609; RRID: AB_393778
Anti-Neurofilament-L, rabbit polyclonal	Cell Signaling	Cat#2837; RRID: AB_823575
Anti-Synaptophysin (SYP), mouse monoclonal	Proteintech	Cat#67864-1-Ig; RRID: AB_2918622
Anti-Vimentin (VIM), rabbit polyclonal	Proteintech	Cat# 10366-1-AP; RRID: AB_2273020
Anti-Vimentin (VIM), mouse polyclonal	Santa Cruz Biotechnology	Cat# sc-373717; RRID: AB_10917747
Anti-TH, rabbit polyclonal	Proteintech	Cat# 25859-1-AP; RRID: AB_2716568
Anti-β-Tubulin III antibody produced in mouse (β3 Tubulin), mouse monoclonal	Sigma-Aldrich	Cat# T8660; RRID: AB_477590
Sheep anti-Mouse IgG ECL Antibody, HRP Conjugated	Cytiva	Cat# NA9310; RRID: AB_772193
Donkey anti-Rabbit IgG, Whole Ab ECL Antibody, HRP Conjugated	Cytiva	Cat# NA934; RRID: AB_772206
Goat anti-Mouse IgG (H + L) Cross-Adsorbed Secondary Antibody, Alexa Fluor™ 488	Thermo Fisher Scientific	Cat# A-11001; RRID: AB_2534069
Goat anti-Rabbit IgG (H + L) Cross-Adsorbed Secondary Antibody, Alexa Fluor™ 594	Thermo Fisher Scientific	Cat# A-11012; RRID: AB_2534079

**Chemicals, peptides, and recombinant proteins**		

DAPI	Thermo Fisher Scientific	Cat# 62248
Neurobasal-A Medium	Gibco	Cat# 10888
Matrigel	Corning	Cat# 356231
Retinoic acid (RA)	Sigma Aldrich	Cat# R2625
Human/Mouse/Rat BDNF Recombinant Protein	PeproTech	Cat# 450-02
B-27™ Supplement (50×)	Gibco	Cat# 17504

**Critical commercial assays**		

miRNeasy Mini Kit	Qiagen	Cat# 217004
QIAshredder	Qiagen	Cat# 79654
SuperScript™ III Reverse Transcriptase	Thermo Fisher Scientific	Cat# 18080
RNase free DNAase Set	Qiagen	Cat# 79254
SYBR™ Green Universal Master Mix	Applied Biosystems	Cat# 4309155

**Deposited data**		

Raw RNA-seq data	This paper	ArrayExpress: E-MTAB-14737
Processed RNA-seq data	This paper	ArrayExpress: E-MTAB-14737
WGS data	See ref.^30^	European Nucleotide Archive: PRJEB45367

**Experimental models: Cell lines**		

CCLF_PEDS_0051_T	DSMZ	Cat# ACC 972
CHP-126	DSMZ	Cat# ACC 304; RRID: CVCL_1123
CHP-134	DSMZ	Cat# ACC 653; RRID: CVCL_1124
GI-ME-N	DSMZ	Cat# ACC 654; RRID: CVCL_1232
IMR-32	DSMZ	Cat# ACC 165; RRID: CVCL_0346
KELLY	DSMZ	Cat# ACC 355; RRID: CVCL_2092
LAN-1	DSMZ	Cat# ACC 655; RRID: CVCL_1827
LAN-2	DSMZ	Cat# ACC 671; RRID: CVCL_1829
LAN-5	DSMZ	Cat# ACC 673; RRID: CVCL_0389
LAN-6	DSMZ	Cat# ACC 674; RRID: CVCL_1363
LS	DSMZ	Cat# ACC 675; RRID: CVCL_2105
MHH-NB-11	DSMZ	Cat# ACC 157; RRID: CVCL_1412
NBL-S	DSMZ	Cat# ACC 656; RRID: CVCL_2136
NGP	DSMZ	Cat# ACC 676; RRID: CVCL_2141
NMB	DSMZ	Cat# ACC 657; RRID: CVCL_2143
SH-SY5Y	DSMZ	Cat# ACC 209; RRID: CVCL_0019
SIMA	DSMZ	Cat# ACC 164; RRID: CVCL_1695
SK-N-BE(2)	DSMZ	Cat# ACC 632; RRID: CVCL_0528

**Oligonucleotides**		

Primers for fusion *CNOT2::TSPAN8*	This paper	N/A
Primers for qPCR	This paper	N/A

**Software and algorithms**		

Trimming	fastq-mcf	ea-utils-bd148d4
Quality control	FastQC	v0.11.9
Alignment	STAR aligner	2.7.5c
Counting	Salmon	1.4.0
Normalization, differential expression	DESeq2	1.44
Functional analysis: Transcription factors analysis including CollecTRI regulon database	decoupleR, CollecTRI	2.10.0
Functional analysis: Gene Ontology	clusterProfiler	4.10.0
Gene ontology database	GO-db	3.18.0
Mutation analysis, pipeline	DSMZ	see ref.^31^
Fusion genes	FusionCatcher	1.33

### Experimental model and study participant details

#### Cell lines and culture conditions

The 18 human NB cell lines were taken from the authenticated and mycoplasma-free stocks of DSMZ cell bank (DSMZ - German Collection of Microorganisms and Cell Cultures GmbH, Braunschweig, Germany). Cell lines were cultured and maintained in proliferation medium under conditions as described in Table S1 and as listed in the catalog page of each cell line at DSMZ website, in humidified atmosphere at 37°C with 5% CO_2_. All experiments in this study were performed with NB cell lines from DSMZ except the WGS dataset (see in Method Details section: Whole Genome Sequencing (WGS) and genomic rearrangement analysis). Full names, tissue origins, donor sex and age were listed in Table S1.

### Method details

#### Neuron differentiation induction

The NB cell line differentiation was performed as described previously.^8^ The 6 well, 24 well cell culture Nunclon Delta plate (Thermo Fisher Scientific), or 4 well PCA chamber (Sarstedt) was coated with Matrigel (Corning) prior to seeding cell at a cell density of 50,000 cells/mL in the proliferation medium. After 24 h the first neuron differentiation stage ND1 was initiated by switching to ND1 medium containing the basal proliferation medium ((DMEM, RPMI 1640, IMDM, or MEM/F12, from Gibco), 2.5% h.i. FBS (Sigma Aldrich), 10 μM Retinoic acid (RA, Sigma Aldrich)). The cells were maintained in ND1 medium for 5 days with fresh medium change every second day. After 5 days in ND1, the second stage neuron differentiation ND2 was initiated by switching to ND2 medium containing the neurobasal-A media (Gibco), 50 ng/mL BDNF (PeproTech), 20 mM KCl (Sigma Aldrich) and 1× B27 (Gibco). The cells were maintained in ND2 medium for 5 days with fresh medium change every second day. The undifferentiated (ND0, day 0) and differentiated (ND1 at day 5, ND2 at day 10) samples were collected for RNA related analysis, by merging 2 wells from a 6 well plate as single sample. All experiments were performed at least three times independently for RNA collection. Cells from 24 well plate or 4 well chamber were fixed for staining. Brightfield images were captured on a Zeiss Axio microscope with Axiocam 208 color to monitor the cell differentiation process.

#### DNA, RNA and protein sample preparation

DNA was extracted using the QIAamp DNA Blood Mini Kit (Qiagen) according to the manufacturers’ instructions.

Total RNA was isolated using the miRNeasy Mini Kit (Qiagen) including DNase digestion with RNase free DNAase Set (Qiagen) following the manufacturer’s instructions.

Protein lysate from 4 × 10^6^ cells of each NB cell line was prepared with Laemmli Sample Buffer (Biorad) supplemented with 2-Mercaptoethanol (Biorad) and protease inhibitors (Roche) and was boiled at 100°C for 10 min prior to analysis by western blot.

#### mRNA-sequencing and expression analysis

Library preparation and mRNA-sequencing (RNA-seq) were performed by Eurofins Genomics as described.^22^ Briefly, strand-specific mRNA-libraries were prepared with the NEBNext Ultra II Directional RNA Library Prep Kit for Illumina (New England Biolabs), amplified and sequenced on a NovaSeq 6000 (Illumina) with 2 × 151 cycles (paired-end) with at least 30 million reads per sample. Insert sizes were aimed at 2 × 151 bp length in order to increase the probability to capture fusion genes and to achieve non-redundant reads for variant calling.^31^^,^^32^

Preprocessing and analysis were conducted as described previously^22^ which is stored at zenodo (https://zenodo.org/records/6401600) and github (https://github.com/claupomm/RNA-seq_ll100/tree/v1.0.0). Briefly, reads were trimmed with fastq-mcf, ea-utils, checked for quality using FastQC, quantified with Salmon, and analyzed with R/Bioconductor package DESeq2 including default normalization and testing along with estimation of size factors, dispersion, and binomial GLM fitting and Wald statistics. For the cluster dendrogram, 80% of the top varying genes were taken, standard normalized for the distance measure, and clustered by the agglomeration method ward.D2. The top 50 differentially expressed genes of group A versus group B were plotted in a heatmap (Figure 2C).

Transcription factor activities were inferred from processed bulk RNA-seq data via decoupleR^33^ on the collection of CollecTRI regulons^34^ following the manual (https://bioconductor.org/packages/3.19/bioc/vignettes/decoupleR/inst/doc/tf_bk.html) except for including DESeq2 statistics and different colors. Explicitly, DESeq2 normalized data were log2-transformed (log2(n+1)), human collectTRI network were selected, activity inference with univariate lineal model (ULM) were run with minsize 5, and the top 25 transcription factors were captured in the heatmap (Figures 2B and 2E). TF activities were inferred from the log2FC, the Wald statistics, and padj of DESeq2, whereby no gene was excluded (barplot, Figure 2F). Target genes for single TFs were visualized in scatterplots (Figures 3B and 3C), based on the Wald statistics of DESeq2 (log2FC, stat, padj).

Gene set enrichment analysis (GSEA) of gene ontology (GO) terms for cellular component (CC)^35^ were found by clusterProfiler^36^ based on log2FC data of DESeq2 and the default filter set on minimal gene set size to 100, maximum gene set size 500, and an adjusted *p*-value cutoff 0.05 (Figure 2D).

Raw RNA-seq fastq files as well as normalized expression data are archived at ArrayExpress: E-MTAB-14737. Gene expression data can be accessed freely and interactively at DSMZCellDive^37^ (https://celldive.dsmz.de/rna/neuroblastoma, released 28 May 2025).

#### Mutation and fusion gene analysis with RNA-seq data

Mutation calling was performed as described previously including most additional filter steps.^31^ In short, after pre-processing the RNA-seq data, mutations were called via GATK HaplotypeCaller and subsequent elaborated filtering processes encompassed RNA edit sites, low complexity regions, low depth, common variants, and non-coding regions. However, the filter of variants occurring in over 20% of the samples was skipped in order to keep recurring mutations. Mutations were visualized in a waterfall plot via the R package GenVisR.^38^ Workflow and scripts are archived at zenodo (https://doi.org/10.5281/zenodo.13759327) and github (https://github.com/claupomm/RNA-seq_snv_tumour_only/tree/v1.0.0).

For identifying and annotating novel and known in-frame fusion events based on split reads in the RNA-seq data of the NB cells, we applied FusionCatcher (v1.30) as described previously^22^ but slightly different thresholds for filtering as fusions were further filtered for only in-frame ones detected with at least one fusion calling algorithms and a minimal split read count of 2. Selected fusion transcripts were confirmed by PCR amplification and Sanger Sequencing.

#### Whole genome sequencing (WGS) and genomic rearrangement analysis

The WGS data for cell lines LS, NGP, GI-ME-N from previous study^30^ was used to analyze the genomic rearrangements. In this dataset, the LS and NGP cell lines were from ATCC - the American Type Culture Collection (ATCC), and GI-ME-N was from DSMZ - German Collection of Microorganisms and Cell Cultures GmbH, Braunschweig. The breakpoints and amplifications in chromosome 12 of LS and NGP cell lines were visualized using IGV browser.

The gene rearrangements were assessed in neuroblastoma cell lines using previously established and complementary approaches as reported by Peifer et al.^39^ In the KELLY cell line, rearrangement of the *TERT* locus was confirmed experimentally by fluorescence *in situ* hybridization (FISH) analysis.

#### Fluorescent telomeric repeat amplification protocol (f-TRAP) assay

The f-TRAP assay was performed as previously described.^40^ This method can detect telomerase activity to assess the immortality of cell lines but it is not for comparative or absolute quantification of telomerase activity.

#### Droplet digital PCR (ddPCR)

The ddPCR assay was performed as previously described.^41^

#### Quantitative PCR (qPCR)

For gene expression analysis the cDNA synthesis was performed with SuperScript III reverse transcriptase (Thermo Fisher Scientific) according to manufacturer’s instructions with random primers. Reactions were carried out via SYBR™ Green Universal Master Mix (Applied Biosystems) in technical triplicate with diluted cDNAs. Primers for target genes as well as housekeeping gene TBP are listed in Table S8. The 2^−ΔΔCT^ method has been used to calculate the relative fold changes. The expression data was normalized using the Ct values of *TBP*. The relative fold change for undifferentiated samples in Figure 3 was normalized to SH-SY5Y. For differentiation evaluation (Figures 5 and 6), all relative fold change was normalized to sample SIMA (ND0), as for the selected genes *SYP*, *TH*, *GRIN1*, and *SLC18A3*, the NB cell lines showed diverse baseline expression but the SIMA (ND0) has relative high expression of the selected genes. To achieve high Ct for reliable baseline expression for cell line with low target gene expression, and to identify not only upregulated but stable and true expression of the target genes, sample SIMA (ND0) was chosen as reference sample. The qPCR was performed with samples from at least three independent experiment and run independently.

#### Western blot

Cell lysates were separated in a 10% Tris-Glycine gel and transferred onto nitrocellulose membranes using a Trans-Blot® SD Semi-Dry Transfer Cell (Biorad) with 0.14 A for 40 min per gel. After blocking with 5% non-fat milk, the membrane was incubated with the primary antibody: anti-N-myc (1:500, rabbit polyclonal, Cell Signaling), anti-GAPDH (1:5,000, mouse monoclonal, Abcam), anti-Synaptophysin (SYP, 1:10,000, mouse monoclonal, Proteintech), anti-Vimentin (E−5) (VIM, 1:10,000, mouse monoclonal, Santa Cruz). In the following step, HRP conjugated secondary antibodies were applied: anti-Mouse IgG (1:1,000, NA9310, Cytiva) an anti-Rabbit IgG (1:1,000, NA934, Cytiva). Proteins were detected by Western-Lightning-ECL (PerkinElmer) in a digital system ChemoStar Imager (INTAS).

#### Immunocytochemistry

Cells seeded in chambers without or with differentiation treatment were fixed in 4% formaldehyde (Carl Roth) for 15 min followed by permeabilization in 0.25% Triton™ X-100 (Sigma Aldrich) for 5 min at room temperature. After incubation in blocking buffer (1% BSA in PBS) for 30 min, primary antibodies were added at the appropriate dilution in blocking buffer to cells for overnight incubation at 4°C. After gently washed in PBS, cells were incubated with secondary antibodies together with DAPI for 1 h at room temperature in the dark. Fluorescent images were captured on a Zeiss Axiovert A1 microscope. Due to the uneven distribution of cells on plate or chamber slides, the over-crowded center was excluded from the imaging due to weak signal and overlapping of cell clusters which were not suitable for counting. Images taken from sides of each slide were used for quantification.

Image processing including merging or separating channels was performed with Fiji software. For cell marker quantification, all images were captured under the same setting and processed using Fiji Cell Counter function for counting positive, negative or total cell numbers. For neurite length calculation, all images were captured under the same settings. Images were first converted to a binary format and skeletonized using Fiji, then analyzed by Fiji plugin Analyze Skeleton 2D/3D to measure the total branch length from the entire image. For each differentiation stage, 6 images captured from two independent experiments (3 images per experiment) were used. The number of nuclei from each image was quantified either with Fiji Analyze Particle function if the nuclei were not clustered or counted manually using Fiji Cell Counter function. The result was presented as total branch length (μm)/nuclei.

Antibodies used for immunostaining: anti-Ki67 (Ki67, 1:300, mouse monoclonal, BD), anti-Neurofilament-L (NF-L, 1:600, rabbit polyclonal, Cell signaling), anti-Synaptophysin (SYP, 1:200, mouse monoclonal, Proteintech), anti-Vimentin (VIM, 1:200, rabbit monoclonal, Proteintech), anti-β-Tubulin III (β3 Tubulin, 1:400, mouse monoclonal, Sigma-Aldrich), anti-TH (1:200, rabbit polyclonal, Proteintech), Alexa Fluor® 448 goat anti-mouse IgG (H + L) and Alexa Fluor® 594 goat anti-rabbit IgG (H + L) (1:200, ThermoFischer Scientific). DAPI (ThermoFischer Scientific) working concentration was 1 μg/mL.

### Quantification and statistical analysis

The RNA-seq data for 18 NB cell lines was obtained from single sample. All gene expression validation experiments (qPCR) were independently reproduced at least three times with samples from more than three independent experiments. Quantification of cell markers and neurite growth from IF images were performed from two independent experiments with at least 3 images from each chamber or slide, and each image must contain more than 30 cells for undifferentiated stage, and more than 50 cells for differentiation stages. Western blot was also performed with two independent experiments. Data are presented as mean ± standard deviation (SD) as specified in the figure legends. The comparison between multiple groups was performed using one-way ANOVA (compares the means of 3 or more groups to determine significant differences) with a normality test (Shapiro-Wilk) followed by Holm-Sidak’s pairwise multiple comparison test using Sigmaplot 11.0. A *p* value <0.05 was considered statistically significant. ∗*p* < 0.05, ∗∗*p* < 0.01, ∗∗∗*p* < 0.001, n.s. no significant difference.

## Supplemental information

Document S1. Figures S1–S7

Table S1. Cell line list, related to STAR methods

Table S2. Mutations of neuroblastoma-related genes, related to Figure 1 and Table 1

Table S3. Fusions in-frame, related to Figure 1 and Table 1

Table S4. Neuron genes and related diseases, related to Figure 1, Figure 6, and Table 1

Table S5. Mutations of neuronal genes, related to Figure 1 and Table 1

Table S6. DEGs of ADRN vs. MES, related to Figure 2 and Figure 3

Table S7. Gesa_go_A_B_analysis, related to Figure 2

Table S8. PCR and qPCR primers, related to STAR methods

## References

[1] Ramesh S., Arachchige A.S.P.M. (2023). Depletion of dopamine in Parkinson’s disease and relevant therapeutic options: A review of the literature. AIMS Neurosci..

[2] H Ferreira-Vieira T., M Guimaraes I., R Silva F., M Ribeiro F. (2016). Alzheimer’s disease: Targeting the Cholinergic System. Curr. Neuropharmacol..

[3] Nimgampalle M., Chakravarthy H., Sharma S., Shree S., Bhat A.R., Pradeepkiran J.A., Devanathan V. (2023). Neurotransmitter systems in the etiology of major neurological disorders: Emerging insights and therapeutic implications. Ageing Res. Rev..

[4] Chen S., Xu D., Fan L., Fang Z., Wang X., Li M. (2022). Roles of N-Methyl-D-Aspartate Receptors (NMDARs) in epilepsy. Front. Mol. Neurosci..

[5] Cetin S., Knez D., Gobec S., Kos J., Pišlar A. (2022). Cell models for Alzheimer’s and Parkinson’s disease: At the interface of biology and drug discovery. Biomed. Pharmacother..

[6] Goettert M., Hoffmann L., Martins A., Majolo F., Contini V., Laufer S. (2023). Neural regeneration research model to be explored: SH-SY5Y human neuroblastoma cells. Neural Regen. Res..

[7] Xicoy H., Wieringa B., Martens G.J.M. (2017). The SH-SY5Y cell line in Parkinson’s disease research: a systematic review. Mol. Neurodegener..

[8] Dravid A., Raos B., Svirskis D., O’Carroll S.J. (2021). Optimised techniques for high-throughput screening of differentiated SH-SY5Y cells and application for neurite outgrowth assays. Sci. Rep..

[9] Targett I.L., Crompton L.A., Conway M.E., Craig T.J. (2024). Differentiation of SH-SY5Y neuroblastoma cells using retinoic acid and BDNF: a model for neuronal and synaptic differentiation in neurodegeneration. In Vitro Cell. Dev. Biol. Anim..

[10] Ducray A.D., Wiedmer L., Herren F., Widmer H.R., Mevissen M. (2020). Quantitative characterization of phenotypical markers after differentiation of SH-SY5Y cells. CNS Neurol. Disord. - Drug Targets.

[11] Lopes F.M., Da Motta L.L., De Bastiani M.A., Pfaffenseller B., Aguiar B.W., De Souza L.F., Zanatta G., Vargas D.M., Schönhofen P., Londero G.F. (2017). RA differentiation enhances dopaminergic features, changes redox parameters, and increases dopamine transporter dependency in 6-Hydroxydopamine-Induced neurotoxicity in SH-SY5Y cells. Neurotox. Res..

[12] Martin E.R., Gandawijaya J., Oguro-Ando A. (2022). A novel method for generating glutamatergic SH-SY5Y neuron-like cells utilizing B-27 supplement. Front. Pharmacol..

[13] Bayeva N., Coll E., Piskareva O. (2021). Differentiating Neuroblastoma: A systematic review of the retinoic acid, its derivatives, and synergistic interactions. J. Personalized Med..

[14] Meeser A., Bartenhagen C., Werr L., Hellmann A.M., Kahlert Y., Hemstedt N., Nürnberg P., Altmüller J., Ackermann S., Hero B. (2022). Reliable assessment of telomere maintenance mechanisms in neuroblastoma. Cell Biosci..

[15] Ackermann S., Cartolano M., Hero B., Welte A., Kahlert Y., Roderwieser A., Bartenhagen C., Walter E., Gecht J., Kerschke L. (2018). A mechanistic classification of clinical phenotypes in neuroblastoma. Science.

[16] Szymansky A., Kruetzfeldt L.M., Heukamp L.C., Hertwig F., Theissen J., Deubzer H.E., Willing E.M., Menon R., Fuchs S., Thole T. (2021). Neuroblastoma Risk Assessment and Treatment Stratification with Hybrid Capture-Based Panel Sequencing. J. Personalized Med..

[17] Boeva V., Louis-Brennetot C., Peltier A., Durand S., Pierre-Eugène C., Raynal V., Etchevers H.C., Thomas S., Lermine A., Daudigeos-Dubus E. (2017). Heterogeneity of neuroblastoma cell identity defined by transcriptional circuitries. Nat. Genet..

[18] Van Groningen T., Koster J., Valentijn L.J., Zwijnenburg D.A., Akogul N., Hasselt N.E., Broekmans M., Haneveld F., Nowakowska N.E., Bras J. (2017). Neuroblastoma is composed of two super-enhancer-associated differentiation states. Nat. Genet..

[19] Wang L., Tan T.K., Durbin A.D., Zimmerman M.W., Abraham B.J., Tan S.H., Ngoc P.C.T., Weichert-Leahey N., Akahane K., Lawton L.N. (2019). ASCL1 is a MYCN- and LMO1-dependent member of the adrenergic neuroblastoma core regulatory circuitry. Nat. Commun..

[20] Wang L., Tan T.K., Kim H., Kappei D., Tan S.H., Look A.T., Sanda T. (2023). ASCL1 characterizes adrenergic neuroblastoma via its pioneer function and cooperation with core regulatory circuit factors. Cell Rep..

[21] Gautier M., Thirant C., Delattre O., Janoueix-Lerosey I. (2021). Plasticity in neuroblastoma cell identity defines a Noradrenergic-to-Mesenchymal transition (NMT). Cancers.

[22] Pommerenke C., Nagel S., Haake J., Koelz A.L., Christgen M., Steenpass L., Eberth S. (2024). Molecular Characterization and Subtyping of Breast Cancer Cell Lines Provide Novel Insights into Cancer Relevant Genes. Cells.

[23] Zimmerman M.W., Durbin A.D., He S., Oppel F., Shi H., Tao T., Li Z., Berezovskaya A., Liu Y., Zhang J. (2021). Retinoic acid rewires the adrenergic core regulatory circuitry of childhood neuroblastoma. Sci. Adv..

[24] Sansevrino R., Hoffmann C., Milovanovic D. (2023). Condensate biology of synaptic vesicle clusters. Trends Neurosci..

[25] Liang J., Tong P., Zhao W., Li Y., Zhang L., Xia Y., Yu Y. (2014). The REST Gene Signature Predicts Drug Sensitivity in Neuroblastoma Cell Lines and Is Significantly Associated with Neuroblastoma Tumor Stage. Int. J. Mol. Sci..

[26] Westerhout E.M., Hamdi M., Stroeken P., Nowakowska N.E., Lakeman A., Van Arkel J., Hasselt N.E., Bleijlevens B., Akogul N., Haneveld F. (2021). Mesenchymal-Type neuroblastoma cells escape ALK inhibitors. Cancer Res..

[27] Zeineldin M., Patel A.G., Dyer M.A. (2022). Neuroblastoma: When differentiation goes awry. Neuron.

[28] Ferguson K.M., Gillen S.L., Chaytor L., Poon E., Marcos D., Gomez R.L., Woods L.M., Mykhaylechko L., Elfari L., Martins da Costa B. (2023). Palbociclib releases the latent differentiation capacity of neuroblastoma cells. Dev. Cell.

[29] Krishna A., Biryukov M., Trefois C., Antony P.M., Hussong R., Lin J., Heinäniemi M., Glusman G., Köglsberger S., Boyd O. (2014). Systems genomics evaluation of the SH-SY5Y neuroblastoma cell line as a model for Parkinson’s disease. BMC Genom..

[30] Rosswog C., Bartenhagen C., Welte A., Kahlert Y., Hemstedt N., Lorenz W., Cartolano M., Ackermann S., Perner S., Vogel W. (2021). Chromothripsis followed by circular recombination drives oncogene amplification in human cancer. Nat. Genet..

[31] Eberth S., Koblitz J., Steenpaß L., Pommerenke C. (2025). Refined variant calling pipeline on RNA-seq data of breast cancer cell lines without matched-normal samples. BMC Res. Notes.

[32] Pommerenke C., Geffers R., Bunk B., Bhuju S., Eberth S., Drexler H.G., Quentmeier H. (2016). Enhanced whole exome sequencing by higher DNA insert lengths. BMC Genom..

[33] Badia-I-Mompel P., Vélez Santiago J., Braunger J., Geiss C., Dimitrov D., Müller-Dott S., Taus P., Dugourd A., Holland C.H., Ramirez Flores R.O., Saez-Rodriguez J. (2022). decoupleR: ensemble of computational methods to infer biological activities from omics data. Bioinform. Adv..

[34] Müller-Dott S., Tsirvouli E., Vazquez M., Ramirez Flores R.O., Badia-I-Mompel P., Fallegger R., Türei D., Lægreid A., Saez-Rodriguez J. (2023). Expanding the coverage of regulons from high-confidence prior knowledge for accurate estimation of transcription factor activities. Nucleic Acids Res..

[35] Aleksander S.A., Balhoff J., Carbon S., Cherry J.M., Drabkin H.J., Ebert D., Feuermann M., Gaudet P., Harris N.L., Hill D.P. (2023). The Gene Ontology knowledgebase in 2023. Genetics.

[36] Yu G., Wang L.G., Han Y., He Q.Y. (2012). clusterProfiler: an R Package for Comparing Biological Themes Among Gene Clusters. OMICS A J. Integr. Biol..

[37] Koblitz J., Dirks W.G., Eberth S., Nagel S., Steenpass L., Pommerenke C. (2022). DSMZCellDive: Diving into high-throughput cell line data. F1000Res..

[38] Skidmore Z.L., Wagner A.H., Lesurf R., Campbell K.M., Kunisaki J., Griffith O.L., Griffith M. (2016). GenViSR: Genomic Visualizations in R. Bioinformatics.

[39] Peifer M., Hertwig F., Roels F., Dreidax D., Gartlgruber M., Menon R., Krämer A., Roncaioli J.L., Sand F., Heuckmann J.M. (2015). Telomerase activation by genomic rearrangements in high-risk neuroblastoma. Nature.

[40] Fähnrich S., Wedemann A., Steenpass L., Dirks W.G. (2024). Optimized for routine: highly sensitive fluorescent Telomeric Repeat Amplification Protocol (f-TRAP). Biotechniques.

[41] Lodrini M., Sprüssel A., Astrahantseff K., Tiburtius D., Konschak R., Lode H.N., Fischer M., Keilholz U., Eggert A., Deubzer H.E. (2017). Using droplet digital PCR to analyze MYCN and ALK copy number in plasma from patients with neuroblastoma. Oncotarget.

