# Supplementary material for: Neuronal differentiation of neuroblastoma cell lines for neurological disease modeling
Source: iScience. 2026 Jun 18;29(7):116469. doi: 10.1016/j.isci.2026.116469 (PMC13312023; doi:10.1016/j.isci.2026.116469)
Supplement: Document S1. Figures S1–S7 [file mmc1.pdf]

iScience, Volume *29*

Supplemental information

Neuronal differentiation of neuroblastoma cell lines for neurological disease modeling

Claudia Pommerenke, Vivien Hauer, Sonja Eberth, Lisa Werr, Hannah Kallnischkies, Ulfert Rand, Stefan Nagel, Christoph Bartenhagen, Wilhelm Gerhard Dirks, Matthias Fischer, Laura Steenpass, and Haicui Wang

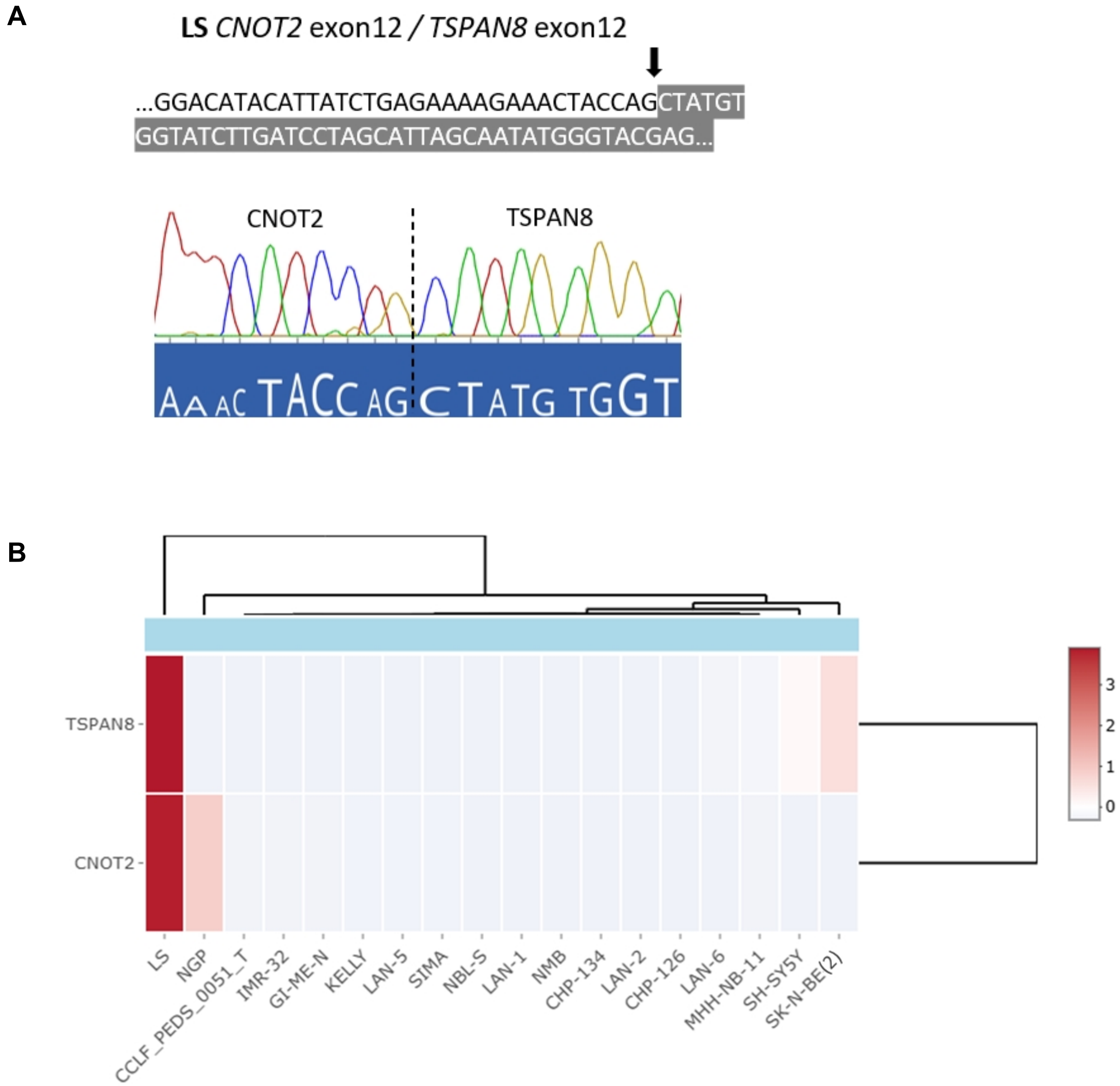

Figure S1 The gene fusion detected in RNA-seq data. Related to Table 1.

(A) One predicted fusion gene *CNOT2*::*TSPAN8* in LS was confirmed by Sanger sequencing. (B) The fusion gene resulted in high expression of both genes. The heatmap of genes *TSPAN8* and *CNOT2* was generated from DSMZCellDive for 18 NB cell lines.

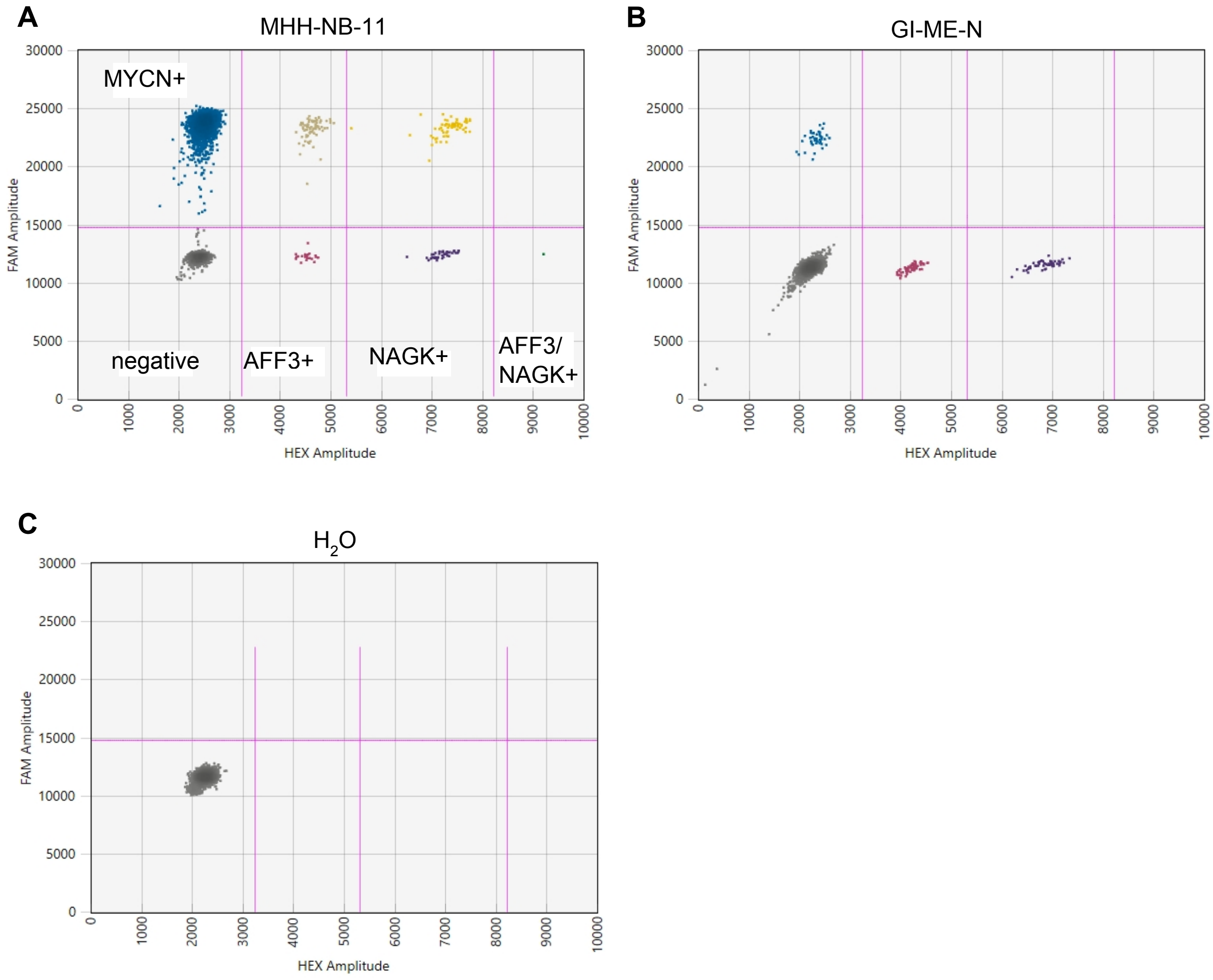

Figure S2 *MYCN* copy number detected by ddPCR from genomic DNA. Related to Table 1.

(A) *MYCN* amplified cell line and (B) *MYCN* not amplified cell line with (C) water control were used to establish the method. The genes *AFF3* (*AF3/MLLT3* fusion gene) and *NAGK* (N-acetylglucosamine kinase) are frequently used as normal diploid reference genes for copy number variation (CNV) analysis in droplet digital PCR (ddPCR) assays.

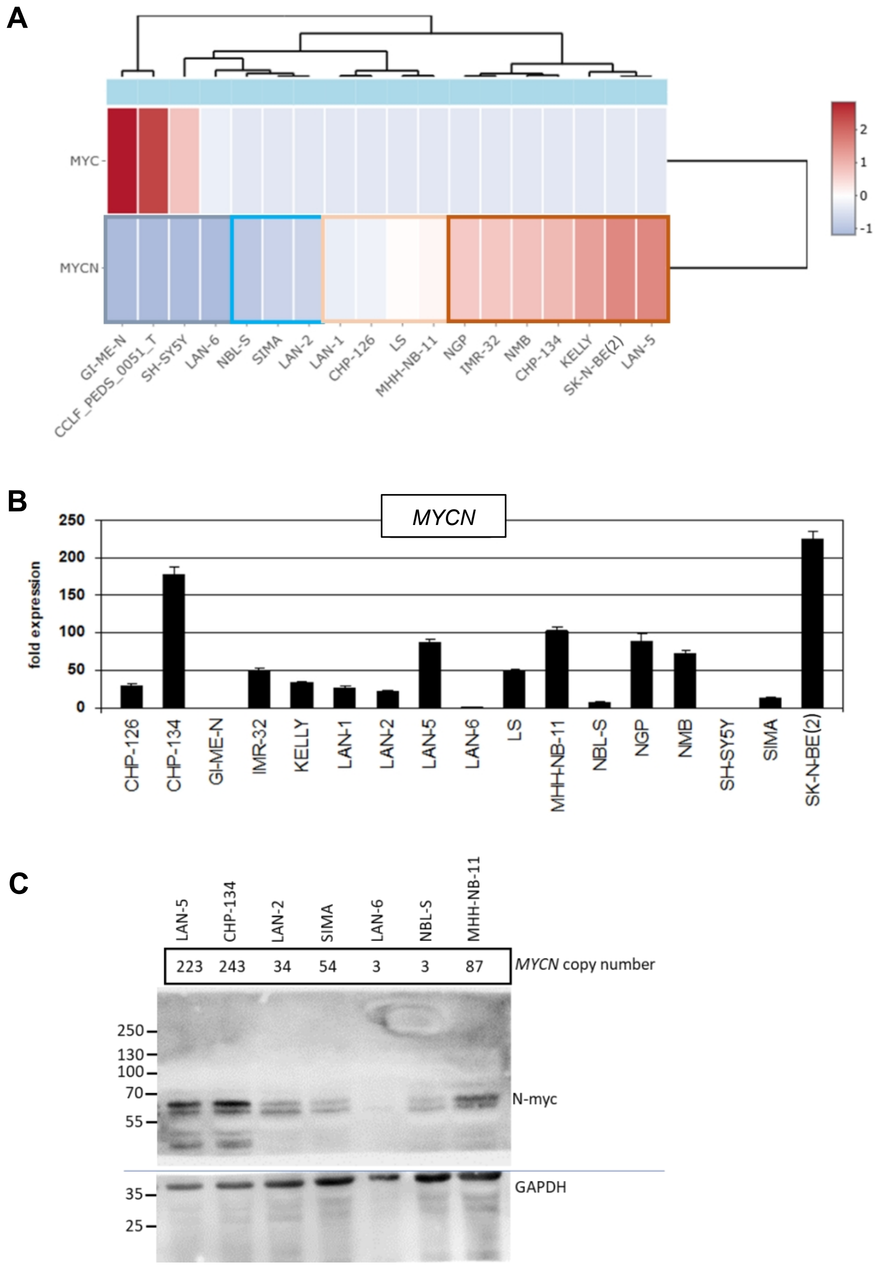

Figure S3 *MYCN* expression in NB cell lines. Related to Table 1.

(A) The *MYCN* gene expression detected in RNA-seq. The heatmap for *MYCN* and *MYC* (c-myc) gene expression was generated with DSMZCellDive. From low to high, the expression of *MYCN* gene was scored in four groups (0, +, ++, +++), as listed in Table 1. (B) *MYCN* expression in NB cell lines detected by qPCR. (C) The N-myc protein expression detected by western blot. Membrane was cut into two pieces to blot with anti N-myc or anti-GAPDH antibodies respectively. Results confirmed LAN-5 with highest N-myc level corresponding to highest *MYCN* mRNA level (+++) and MHH-NB-11 with a N-myc level lower than LAN-5 (++) but higher than NBL-S (+).

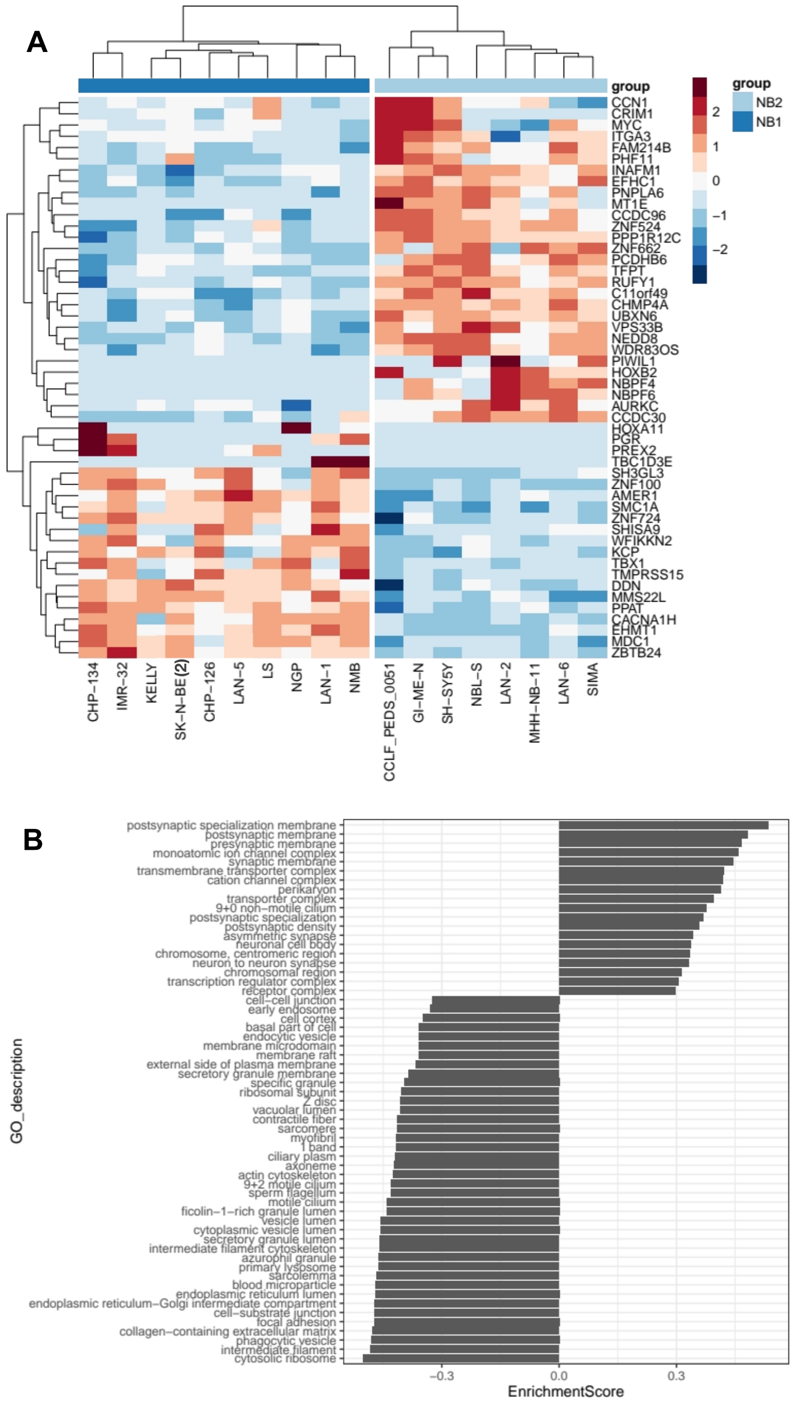

Figure S4 Differential expression of genes driven by *MYCN* gene. Related to Figure 2.

(A) The differentially expressed genes (DEGs) in NB1 and NB2 groups. NB1 correlates to *MYCN* high expression and NB2 correlates to *MYCN* low expression including the line CCLF_PEDS_0051. Only top 50 DEGs were showed. (B) Gene Set Enrichment Analysis (GSEA) on Gene Ontology (GO) between NB1 and NB2 groups.

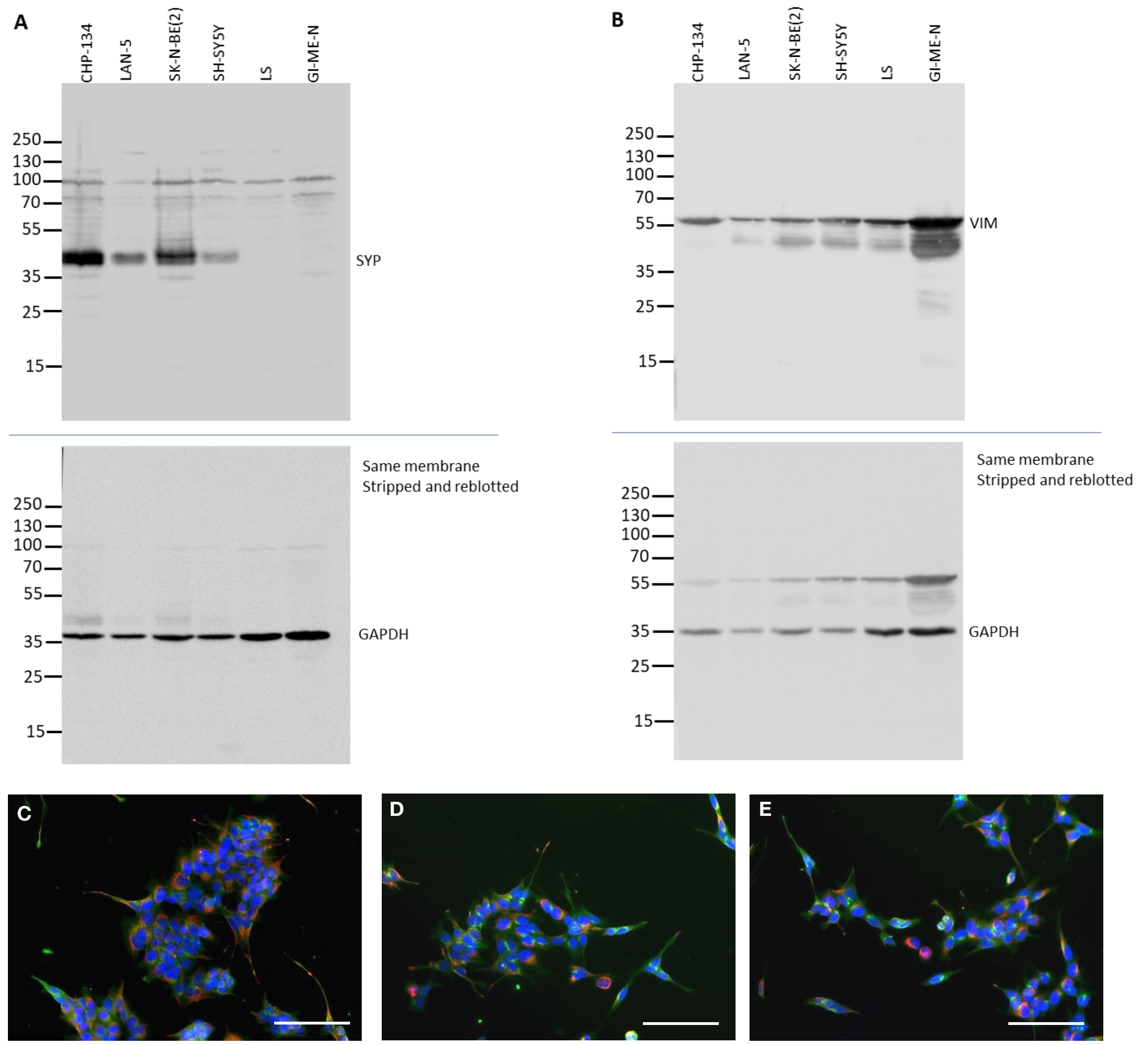

Figure S5 Expression of selected MES-type marker vimentin (VIM) and ADRN-type marker synaptophysin (SYP). Related to Figure 3.

(A) Western blot in selected NB cell lines using anti-SYP antibody. The molecular weight of SYP is around 39kDa, similar to GAPDH (36kDa). The same membrane was stripped after detection of SYP and used to detect GAPDH. (B) Western blot in selected NB cell lines using anti-VIM antibody. The same membrane was stripped after detection of VIM and used to detect GAPDH. (C-E) Cell morphology of cell line SK-N-BE(2). Cells were stained with ADRN-type marker SYP (green) and MES-type marker VIM (red). Scale Bar: 100μm.

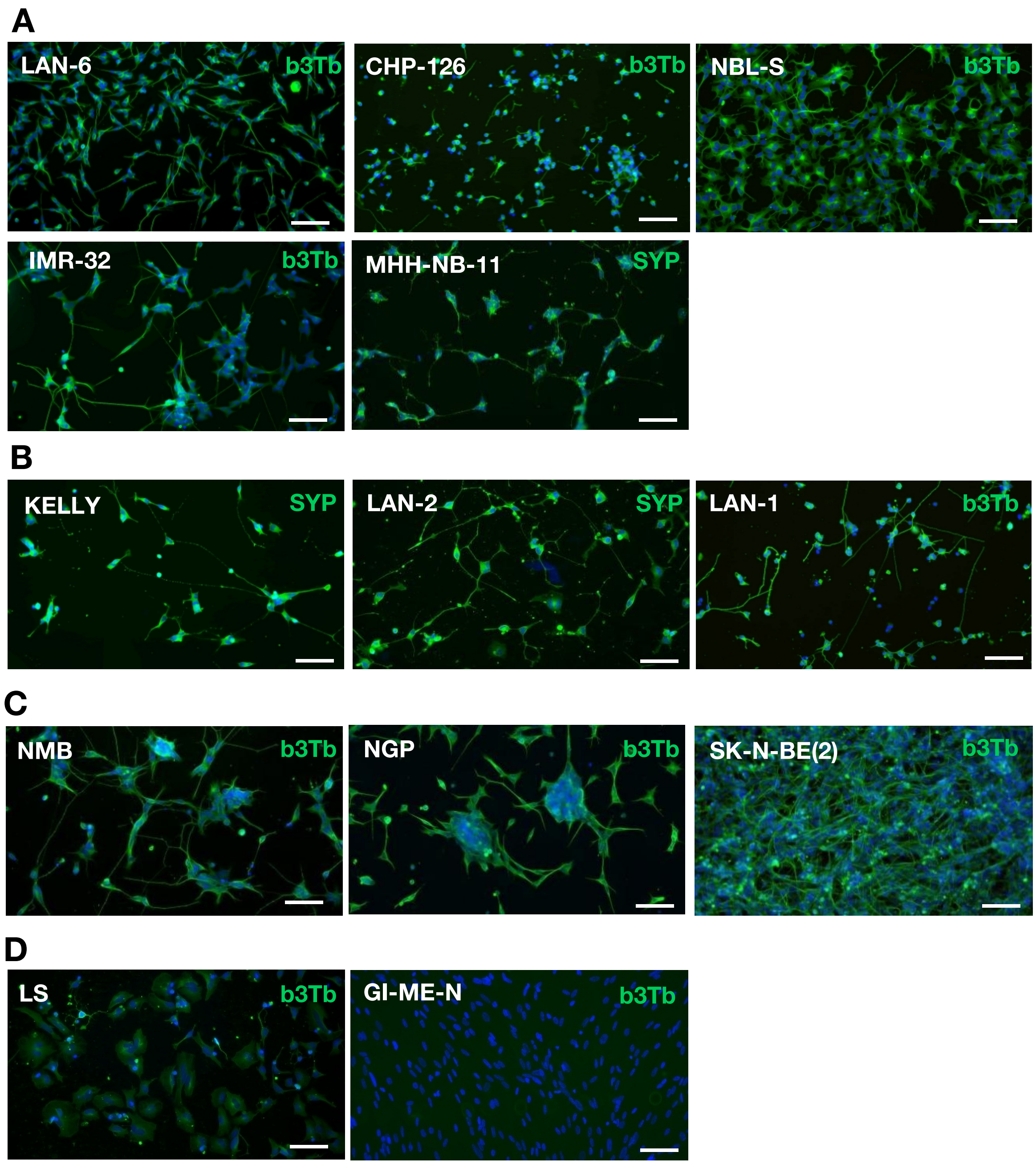

Figure S6 NB cells stained with neuronal markers at ND2 after RA and BDNF treatment. Related to Figure 4 and Figure 5.

The NB cells at ND2, d10 were labeled with neuronal specific markers class III β-tubulin (b3Tb) or synaptophysin (SYP). (A) ADRN-type cell lines poorly responded to RA and BDNF treatment. (B) ADRN-type cell lines with only few survived neuron-like cells. (C) ADRN-type cell lines with clusters after treatment. (D) MES-type cell lines showed no neuronal morphology. Scale Bar: 100μm.

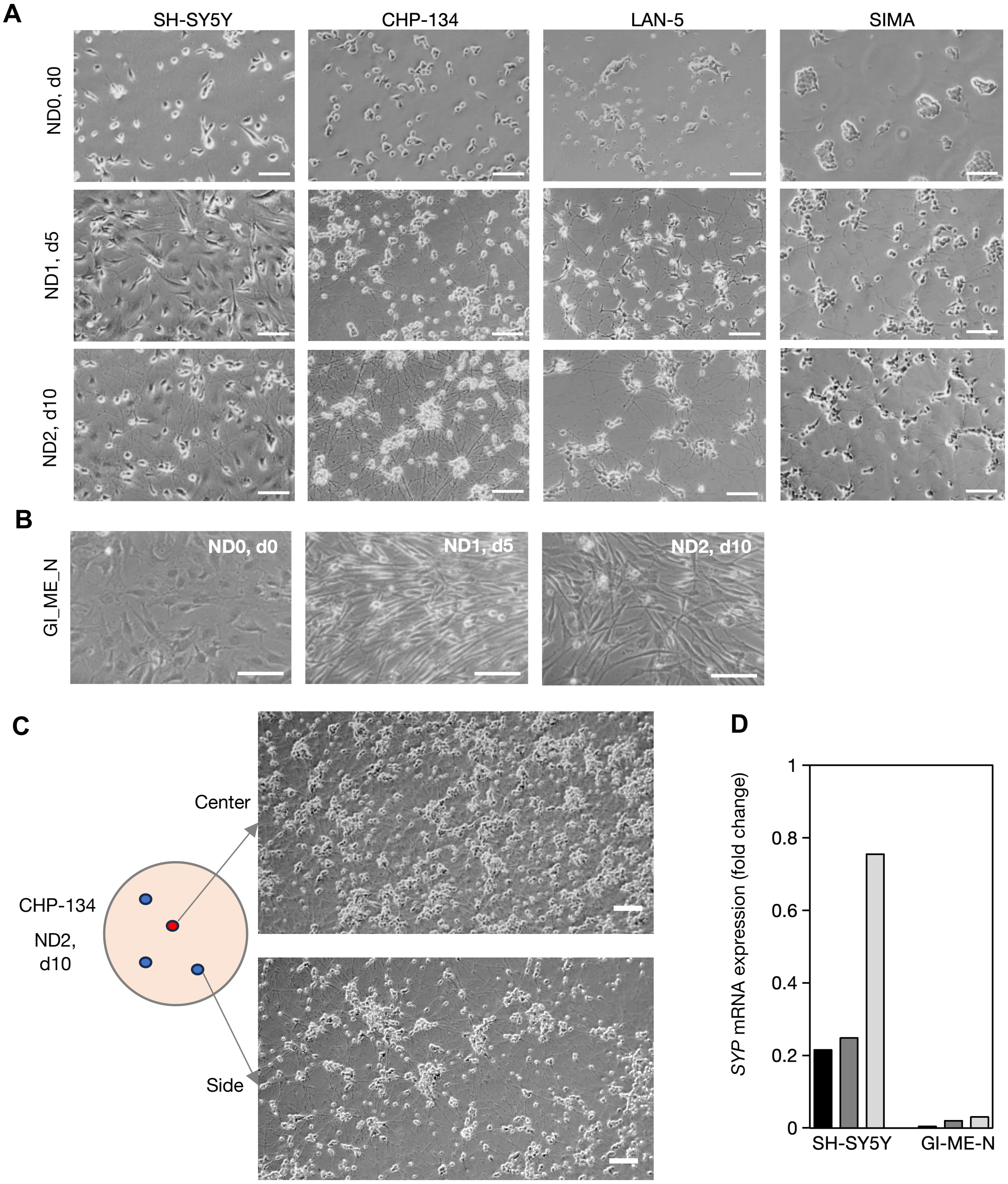

Figure S7 Treatment of NB cell lines with RA and BDNF. Related to Figure 4 and Figure 5.

(A-B) Morphology changes of NB cell lines at three ND stages. Scale Bar: 100μm. (C) Illustration of unevenly distributed neuron-like cells from one well of CHP-134 after differentiation treatment. The center contained highly condensed neuron-like cells which also often detached from the surface, while the side or the edge had more spread cells. Bar: 100μm. (D) *SYP* gene expression at three ND stages in cell line SH-SY5Y and GI-ME-N. The relative fold change detected by qPCR in different NB cell lines was reported as the level of mRNA relative to the SIMA cell line of undifferentiated stage (ND0, d0).
